# Microbes, macrophages, and melanin: a unifying theory of disease as exemplified by cancer

**DOI:** 10.3389/fimmu.2024.1493978

**Published:** 2025-02-06

**Authors:** Stacie Z. Berg, Jonathan Berg

**Affiliations:** Department of Translational Biology, William Edwards LLC, Baltimore, MD, United States

**Keywords:** cancer, pathogens, tumor, bacteria & fungi, biofilm, microorganisms, subclinical infection, infection - immunology

## Abstract

It is widely accepted that cancer mostly arises from random spontaneous mutations triggered by environmental factors. Our theory challenges the idea of the random somatic mutation theory (SMT). The SMT does not fit well with Charles Darwin’s theory of evolution in that the same relatively few mutations would occur so frequently and that these mutations would lead to death rather than survival of the fittest. However, it would fit well under the theory of evolution, if we were to look at it from the vantage point of pathogens and their supporting microbial communities colonizing humans and mutating host cells for their own benefit, as it does give them an evolutionary advantage and they are capable of selecting genes to mutate and of inserting their own DNA or RNA into hosts. In this article, we provide evidence that tumors are actually complex microbial communities composed of various microorganisms living within biofilms encapsulated by a hard matrix; that these microorganisms are what cause the genetic mutations seen in cancer and control angiogenesis; that these pathogens spread by hiding in tumor cells and M2 or M2-like macrophages and other phagocytic immune cells and traveling inside them to distant sites camouflaged by platelets, which they also reprogram, and prepare the distant site for metastasis; that risk factors for cancer are sources of energy that pathogens are able to utilize; and that, in accordance with our previous unifying theory of disease, pathogens utilize melanin for energy for building and sustaining tumors and metastasis. We propose a paradigm shift in our understanding of what cancer is, and, thereby, a different trajectory for avenues of treatment and prevention.

## 1 Introduction

Despite 250 years of research (1) and billions of dollars invested in it – between 2016 and 2020 alone, the global investment in cancer research was estimated at about $24.5 billion (2) – cancer remains a global and urgent international health problem. In 2020, there were nearly 10 million cancer deaths (3). Cancer is conventionally thought to be caused by random genetic mutations. We propose here a paradigm shift in our understanding of what cancer is, and thereby a different trajectory for avenues of treatment, prevention and, likely, even a cure, as seen with most infections, as we theorize that cancer is not caused by random mutations but rather is a targeted strategy used by certain microorganisms to survive and thrive. These microbes may be acutely infectious, subclinically infectious, or non-infectious, microbiome-associated, and hereafter, we frequently refer to them collectively as pathogens due to their tendency to kill the host. We posit there are three levels of increasingly complex microbial organization – colonies, biofilms, and tumors. In this third level, the tumor, the pathogens have penetrated the host tissue and immune cells and taken over certain controls, a pathogenic hijacking, and are able to build a matrix around the cells, force the cells to proliferate, activate angiogenesis, and, when disturbed, the pathogens, via breakaway tumor cells and phagocytic immune cells, scatter and build more tumors (metastasize). Whether the spread is through individual pathogens or small communities may explain the timeline differences in observing metastasis, remission after treatment where pathogens are targeted via chemotherapy and other anti-cancer treatments, and, subsequently, aggressive disease, or, it may be due to dormancy of the pathogens after exposure to treatments. Our theory could be used to explain familial and hereditary cancers, as well, through the passing down *in utero* of subclinical infection or plasmids to offspring, causing the same genetic mutations related to a cancer diagnosed in a parent or grandparent. Another avenue may be shared exposures. Families/households share the same microbiomes by eating the same foods and by sharing home environments (4) as well as clinical and subclinical infections, all of which can be passed from generation to generation in shared households. We further posit that mild immune system response, for example, fever, seen in some individuals with cancer is not an immune system response to mutated self cells that have become differentiated enough to be recognized by the immune system, but instead the immune system has detected pathogens, as it does sometimes with pathogens protected in a biofilm, without recognition of the immensity of the community.

We have identified several characteristics in cancerous tumors: multiple genetic mutations directed by pathogens, a diverse microbiome made primarily of pathogens; high cysteine levels, strongly suggesting tumors are fueled by pheomelanin; immune system evasion via pathogenic control; and pathogens hiding in and spreading via phagocytes. After identifying these characteristics, we predicted that, with the exception of the first or earliest mutations, the characteristics of benign tumors would be the opposite – mostly commensal bacteria (5), less diverse microbiota with fewer pathogenic bacteria (6), lower in cysteine (7), and lower in tumor (bacteria) recruitment of tumor-associated macrophages (TAMs) (8), and significantly less angiogenesis (9). The scientific literature supports our predictions (5–9). We also predict that the characteristics present in malignant tumors could be present in benign tumors, depending on the specific mutations that have taken place at the point of biopsy, which studies suggest to be the case (10, 11). It is noteworthy that benign and malignant tumors share certain risk factors, which we theorize are various forms of energy used by bacteria (12, 13), which we describe in more detail in section 26. We can explain this using our theory: Pathogenic and commensal bacteria can compete for the same nutrients and space. However, commensal bacteria have an advantage in this warfare. They have evolved mechanisms to outcompete pathogens. For example, they are able to produce antimicrobial substances, occupy adhesion sites, and modulate the host’s immune response (14), stimulating the production of regulatory T cell differentiation (15), which are non-phagocytic immune cells, to prevent pathogen colonization (14). This competitive interaction helps protect the host from infections; whereas pathogens are well equipped to live in soil (16), where they will continue to survive, which is, why, we theorize, they are willing to kill the human host, human commensal bacteria are primarily adapted to the stable and nutrient-rich environment of the human body and are therefore, we believe, fighting against pathogens to keep their territory within the human host and keep the human host alive. We theorize that benign tumors are early-stage battlegrounds between commensal and pathogenic microbes where the commensal bacteria, found naturally in the tissue where the benign tumor formed, are able to overtake the pathogens, and where, in rare cases, when benign tumors become more aggressive, pathogens are able to take control, to a degree. As with benign tumors and peptic ulcers, we would expect to find pathogens in any precancerous condition.

**Figure 1:**
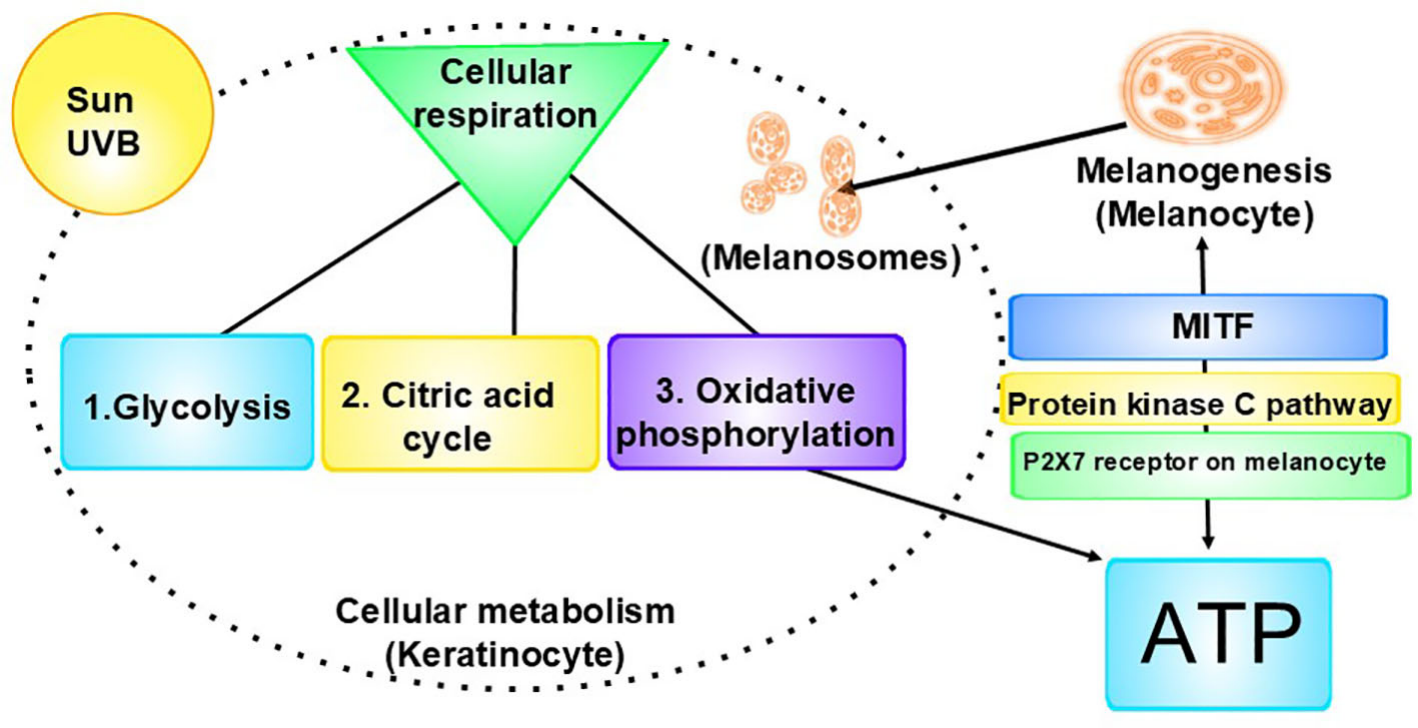
Interrelationship of cellular metabolism, melanogenesis, and ATP production: Left: A key component of cellular metabolism is cellular respiration, the process by which cells make energy (ATP). 1. Glycolysis is the first step of cellular respiration. During glycolysis, one molecule of glucose is broken down into two molecules of pyruvate, producing a small amount of ATP and NADH. 2. Next is the citric acid cycle (Krebs Cycle), during which the pyruvate produced in glycolysis is transported from the cytoplasm into the mitochondria, where it is converted into acetyl-CoA. The acetyl-CoA enters the citric acid cycle, which generates NADH and FADH2 by oxidizing acetyl-CoA. It also produces a small amount of ATP. 3. The final stage is oxidative phosphorylation, where the NADH and FADH2 donate electrons to the electron transport chain, creating a proton gradient across the membrane. ATP synthase uses this gradient to produce ATP from ADP and inorganic phosphate. (Oxygen acts as the final electron acceptor, resulting in H₂O.) Right: ATP is released from cells, including keratinocytes, in response to stimuli, including UV radiation. The released ATP binds to and activates the P2X7 receptors on melanocytes, which leads to the opening of ion channels, allowing the influx of calcium ions (Ca²⁺) and the efflux of potassium ions (K⁺). The increase in intracellular calcium levels can activate the protein kinase C (PKC) pathway. Activation of the PKC pathway leads to the activation of downstream signaling molecules, such as the cAMP response element-binding protein, which in turn can enhance the expression of MITF. MITF is a key regulator of melanogenesis, promoting the production of melanin in melanocytes. The pathway has feedback mechanisms, creating a regulatory loop. This pathway highlights the complex interplay between extracellular signals (in this case, ATP), receptor activation (P2X7), intracellular signaling cascades (PKC), and the biological outcome, melanin (21, 22).

Our theory on cancer being complex microbial, mostly pathogenic, communities is based on the following: 1) statistics do not support the random mutation theory, as out of more than 3 billion nucleotide pairs in human DNA, relatively few, specific mutations are involved in cancer, the mutations occur only in particular regions of certain genes, for example, in 14%-16.8% of all cancers, 1 of 8 specific base pairs are mutated (17), which is a probability of 8/3,117,275,501 per mutation (18), and there is an “intelligence” about which genes and which pathways are affected, that is, it is not chance; 2) various cancers have been linked with pathogens; 3) various tumor types have been discovered to have microbiomes; 4) these tumor types have been discovered to have signature microbiomes; 5) cancer cells communicate with each other through chemical and electrical signaling, as do microbes in biofilms; 6) anticancer therapies are antimicrobials or anti-phagocytics; 7) pathogens can synthesize hyaluronic acid (19), which, at certain molecular weights, can initiate angiogenesis; 8) in some cancers mitochondria are downregulated (we have previously found an inverse relationship between low mitochondrial functioning and high melanogenesis and evidence that melanin, in addition to ATP, supplies energy to cells) (20), (**Figure 1**) and in some cancers mitochondria are upregulated, both of which suggest increased energy demand and together suggest something, such as a pathogen, is dictating energy supply; 9) common risk factors for developing cancer, for example, smoking, obesity, excessive alcohol intake, radiation exposure, ingestion of carbon (via burnt foods, for example), are various forms of molecular energy, provide microorganisms with the energy they need to carry out their tumor-building functions, and therefore, are triggers for cancer; and 10) the cancer genome exhibits significant heterogeneity across different tumor types and within individual tumors, with no two samples from the same patient being genetically identical, and phylogenetic analyses reveal a branching pattern of tumor evolution, suggesting the presence of a diverse biofilm (23, 24). Our theory also has the benefit of explaining why individuals can be exposed to the same risk factors but not all of them develop cancer: They must be host to certain microbes. The purpose of this paper is to provide evidence from the scientific literature of a causal link between microbes and all cancers via infected cells and reprogrammed macrophages, both fueled by melanin. We believe the significance of our discovery of these linkages in the context of our theories cannot be overemphasized.

## 2 Somatic mutation theory: the numbers do not add up

The nearly universally-accepted theory for what causes cancer is the somatic mutation theory, which is based on the idea that cancer forms from a single cell that has accumulated multiple random genetic mutations (25–27). However, Darwin’s theory of evolution, which is the basis of the somatic mutation theory of cancer, asserts genes mutate with equal probability, and those mutations that benefit the organism’s survival enter into the gene pool (28). However, the genetic mutations that cause cancer do not benefit the individual, that is, they do not provide a reproductive or survival advantage. Further, the odds of any individual getting cancer in their lifetime are remarkably high: 1 in 2, or 50 percent (29), and each cancer type has its own statistic for risk, for example, breast cancer, which is 1:8 in women (30). The fact that an individual’s lifetime risk of developing cancer is 50 percent and that every type of cancer has a specific risk ratio, strongly suggests the genetic mutations are not chance mutations.

The actual random chance of any cell developing cancer is unknown due to the complexity. Estimating the exact probability of a single cell developing into a cancerous cell and, consequently, a tumor, and, subsequently, metastasizing is highly complex due to the number of variables involved. It is generally accepted that environmental factors are necessary (31). (As we discuss later, these environmental factors relate back to carbon, an energy source for pathogens.) However, even including environmental factors could not possibly account for the frequency in which cancer at any stage occurs, as there is vast and varied complexity (for example (32–34)) that would remain unaccounted for. In addition to molecular, epigenetic, and other known factors that further complicate the calculations (and further reduce the likelihood of a cell becoming carcinogenic), there are discoveries we have made and that others have made to consider: (1) If cancer is caused only by random mutations and environmental risk factors, then why do mutations occur relatively frequently to specific genes in specific cells and in the necessary order to produce such high rates of cancer? (2) If cancer is caused only by random mutations and environmental risk factors, why is it that phagocytic cells promote the tumor and non-phagocytic cells continue to operate on behalf of the immune system, as there would be an equally probable chance of all immune cells becoming pro tumor? (3) How does the randomness of the currently accepted, conventional theory explain the consistency of the finely orchestrated, vastly complex biological processes seen in tumorigenesis and metastasis, as just one example, the use of platelets for metastasis (detailed in section 9)? It would take an unimaginable number of random specific mutations to orchestrate routine oncological events so intricate and precise that it is not possible to fully explain the development of cancer by random somatic mutations, even coupled with environmental risk factors. Rather, there appears to be an “intelligence” about which genes and which pathways are affected, along with all of the other biological processes that are involved, that is, it is not chance, as the chance of random mutations leading to the development of cancer is infinitesimally small, whereas the risk of developing cancer is relatively high. It is also important to note here that out of more than 3 billion nucleotide pairs (18), only a relatively few specific mutations are involved in cancer, the mutations occur only in particular regions of certain genes, for example, in 14%-16.8% of all cancers, 1 of 8 specific base pairs are mutated (17), and 7 of these 8 sites are evolutionarily conserved (17), which means bacteria and other microorganisms had time to evolve to mutate these sites in specific cancer-promoting ways, and aflatoxin, a molecule produced by *Aspergillus*, can cause one of these eight mutations (17).

Cancer results from genetic changes from mutations, problems repairing damage to DNA, and integration of genetic codes. Yangyanqiu et al. (35) note that the incorporation of bacterial DNA into the human genome could serve as a cis-regulatory element, influencing the activity of host genes, triggering proto-oncogenes, inhibiting tumor suppressor genes, and regulating pathways associated with cancer. Riley et al. (36) found bacterial DNA in the human somatic genome. The group detected the integrations more frequently in tumors, in RNA more so than DNA, and in the mitochondrial genome more so than the nuclear genome.

We theorize mutations that lead to cancer are due to microorganisms targeting and mutating these base pairs in the particular ways seen in cancer. We emphasize that in order to understand cancer, researchers must look at it from the point of view of pathogens. When pathogens kill their human host, it is not to their detriment; they return to an environment rich in nutrients, the soil. There they can continue to thrive (16). We strongly believe that cancer is not a self-cell problem of the host but rather a hijacking of host cells for the benefit of the pathogens and their complex microbial communities.

## 3 Evidence tumors are complex, organized microbial communities

Linking cancer to pathogens is not new. Discovered in 1911 by Peyton Rous, the Rous sarcoma virus was the first pathogen identified to cause cancer in animals (37). Fifty-three years later, in 1964, the Epstein-Barr virus was the first virus to be linked to cancer in humans, a discovery by Anthony Epstein and Yvonne Barr (38). The first bacterium linked to cancer was *Helicobacter pylori* in 1984 by Barry Marshall and Robin Warren (39), which was met with skepticism until Marshall’s famous self experiment (40). In 1994, the liver fluke, *Opisthorchis viverrini*, became the first parasite linked to cancer (41). Certain fungi have been implicated in cancer development; however, no specific fungus has been definitively identified as a primary cause of cancer (42), the reasons for which are explained by our theory herein.

First, we begin with the fact that cancer is not one disease. Malignant tumors exhibit significant diversity, encompassing 250 clinico-pathological types. Furthermore, and we believe this to be critical, within the same tumor, cells exhibit phenotypic, morphologic, and genetic heterogeneity (43). This diversity can be explained using our theory by understanding that the various genetic mutations are being directed by different pathogens. This would result in different mutations in the host cells, even within the same tumor, as biofilms, which is where microorganisms live within hosts, have microbial subcommunities within the greater biofilm community, as discussed later. The fact that each cell is heterogeneous within each tumor provides evidence that it is not one cell, or even several cells, replicating. We theorize it is a diverse group of pathogens infecting cells that are replicating within these cells to form a tumor community, a three-dimensional complex biofilm. We propose the intratumoral microbiota contributes to the initiation and progression of cancer via (1) mutating DNA; (2) activating oncogenic pathways; (3) hijacking of phagocytic cells; (4) initiating metastasis; (5) decreasing antitumor immune responses; (6) promoting cancer progression via upregulating reactive oxygen species (ROS) (which we theorize is the upregulation of melanogenesis for energy) (20) and other strategies, including promoting immunosuppression; and (7) regulating cancer cell physiology and the host immune response via signaling pathways. It should be noted that it is estimated that less than 1% of bacteria are able to be cultured in the laboratory (16).

Nejman et al. (46) analyzed the tumor microbiome of 1,526 tumors from breast, bone, ovarian, lung, pancreatic, and brain cancers, along with adjacent normal tissues. They found each tumor type had distinct microbiome compositions. The bacteria were mostly intracellular and were found in both the cancer cells and immune cells. (In sections 8 and 9 this paper, we will explain the significance to our theory of the immune cell occupation.) Importantly, from our perspective, the intratumor bacteria identified had functions correlated with biological surroundings, that is, they fed off of the contaminants – the known risk factors – of the tissues. In non-small-cell lung cancer, there was a high prevalence of heterogeneous bacteria, which the authors speculate may have come from the tobacco plants, that are able to utilize the chemicals from cigarette smoke metabolites and biosynthesize metabolites used by plants. They had similar findings in breast cancer subtypes. In fact, they found that subtypes of the same tumor type, for example, in breast tumors, estrogen receptor (ER), progesterone receptor (PR), and HER2 subtypes each had a distinct microbiome. In ER+ breast tumors, which have increased oxidative stress compared with ER- breast tumors, they found enriched pathways in bacteria for arsenate detoxification and mycothiol biosynthesis. Arsenic exposure is a risk factor for this subtype of breast cancer. Bacteria have been shown to use mycothiol to detoxify ROS (44). (In section 26 of this paper, we will explain the significance to our theory of risk factors, in fact, being energy for microorganism growth).

As further evidence, recent research in colorectal cancer revealed “a striking association between specific host microbes and aberrant DNA methylation.” Only certain histone regions were affected, and tumors with *Fusobacterium* overgrowth had unique genetic and epigenetic profiles (45). The microbial growth was impressive. More than 1,000 colonies were grown from each of four tumors. Within the 474 representative colonies from five tumors, there were 37 bacterial species. The team found live bacteria from three phyla (*Actinobacteria*, *Firmicutes*, *Proteobacteria*) in breast tumors (46). The group reported that they could not identify the bacteria at the species level for 105 of the colonies. We believe this is further evidence that microbes have been overlooked in tumors, in part because they are difficult to identify due to low biomass (46) and in part because tumors are not typically tested for presence of pathogens.

In addition to viral and bacterial infections, fungal infections, too, have been linked with an increased risk of developing cancer. Dohlman et al. (47) analyzed data from The Cancer Genome Atlas and found disease-related fungi in tumors of the breast, lungs, gastrointestinal tract, and head and neck. *Candida albicans* infections are also associated with an increased risk of cancer and are able to promote cancer progression (48, 49). Narunsky-Haziza and colleagues (50) examined 17,401 patient blood, tissue, and plasma samples of 35 cancer types and found fungal DNA, often within cells, in all 35 types and frequently in macrophages. Extracellular fungal cells were also found, but rarely. (The authors noted that there is no staining method that is capable of detecting all fungi in tissues.) Microbial community compositions differed among cancer types. Intratumoral fungi communities from these treatment-naive tumors were generally permissive with the intratumoral bacterial communities. In contrast, the gut, particularly under anti-cancer or antibiotic therapies, has an antagonistic phenotype, as fungi and bacteria compete for shared resources. Other research demonstrates that within tumors, fungi and bacteria interact by cell-to-cell contact, quorum sensing via the secretion of small molecules, changes in pH, metabolic byproducts, and altering host responses (51).

While relatively little is known about fungi in biofilms and tumors, more is known about fungi and their interactions and roles in the wild. In forests, there are vast networks of fungi in the soil. Mycorrhizal fungi play a major role in trees communicating with each other over long distances (52). Mycorrhizal fungi interact with bacteria in the soil’s rhizosphere (53), and these bacteria interact with the root system’s microbial communities (54) and so serves as a communications hub. The root-associated microbiomes are often referred to as a plant’s secondary genome, because rhizobacteria synthesize molecules that can modify certain traits of the host plant and can enhance plant growth and development (54), much like we theorize pathogens are behaving in tumors. Trees and fungi also exchange nutrients, defense signaling, and allelochemicals (52), similar to what is known to occur between distant biofilms in humans (55).

This ancient ecosystem in forests reveals how fungi and microbes can both direct and be influenced by their host organism, in this case trees, drawing parallels to how we theorize tumor microbial communities interact with their human hosts, typically living symbiotically but sometimes acting to their benefit only, as they have billions of years of evolutionary advantage over trees to have evolved strategies to survive and thrive, even at the expense of their host, as there is no cost to the parasitic behavior; if they kill their host, parasites will return to the nutrient-rich soil and continue to survive. Therefore, we used the forest to conceptualize how fungi and microorganisms work within their host ecosystem for the purpose of gaining insight into how they may behave in human hosts. We likened the soil to tissue, the fungi to the neuronal or other communication networks, and trees to human hosts; trees, like humans, are hosts to a diverse array of microorganisms, including fungi, bacteria, archaea (56), viruses, parasites (nematodes), amoebas, oomycetes (57), and protozoa (58). These microorganisms can be found in various parts of the tree – leaves, bark, wood, roots (59), and within cells (60). The interactions between trees and these microorganisms can be complex, both beneficial and harmful, and play a significant role in forest ecosystems’ health (57). Trees also develop tumors, and it is recognized that these tumors are always caused by infection (61), but because plant cells do not move through the tree, and, therefore, cannot metastasize, and trees do not have vital organs like animals, they often survive. Nonetheless, this macroenvironment gives us a window into the interplay between microbiomes and tumors from the perspective of fungi and other microorganisms with the host as a temporary lodging center that will eventually be recycled by the soil microbiome. It is a solely advantageous cycle for the fungi and microbes. From this perspective, cancer in humans from the vantage point of evolution makes sense.

We cannot leave our forest analogy without mentioning carbon sequestration. As carbon sinks, forests illustrate their vital need for carbon and its various uses in the survival of forest life: (1) Trees and plants absorb carbon dioxide from the atmosphere and use it to produce glucose and oxygen via photosynthesis. The glucose is then used as an energy source for growth and development. Microbes also use carbon as an energy source for growth and metabolism (62), so it is not surprising that microbes are key in determining how much carbon is stored in the soil (63). (Later in this article, we will describe how risk factors for cancer, with the exception of infection, are all carbon related, hence, according to our theory, microbe related.) (2) Organic carbon also improves soil structure by enhancing soil aggregation. This leads to better aeration, water retention, and nutrient availability, which, in turn, supports plant growth and microbial activity (62). This structuring appears similar to biofilm and tumor matrices from our vantage point.

Other ancient organisms, including helminths (64–66) and other parasites (66) and archaea (67), are also known to cause cancer in humans. Helminths can influence the immune response in mucosal sites, where biofilms are present, and these interactions can affect the composition and behavior of the biofilm community (68), as can unicellular parasites from within the biofilm (69). Of importance, there are no reports in the scientific literature of true axenic mice – those without any microorganisms – ever developing cancer.

## 4 Both biofilms and tumors have matrices

**Figure 2:**
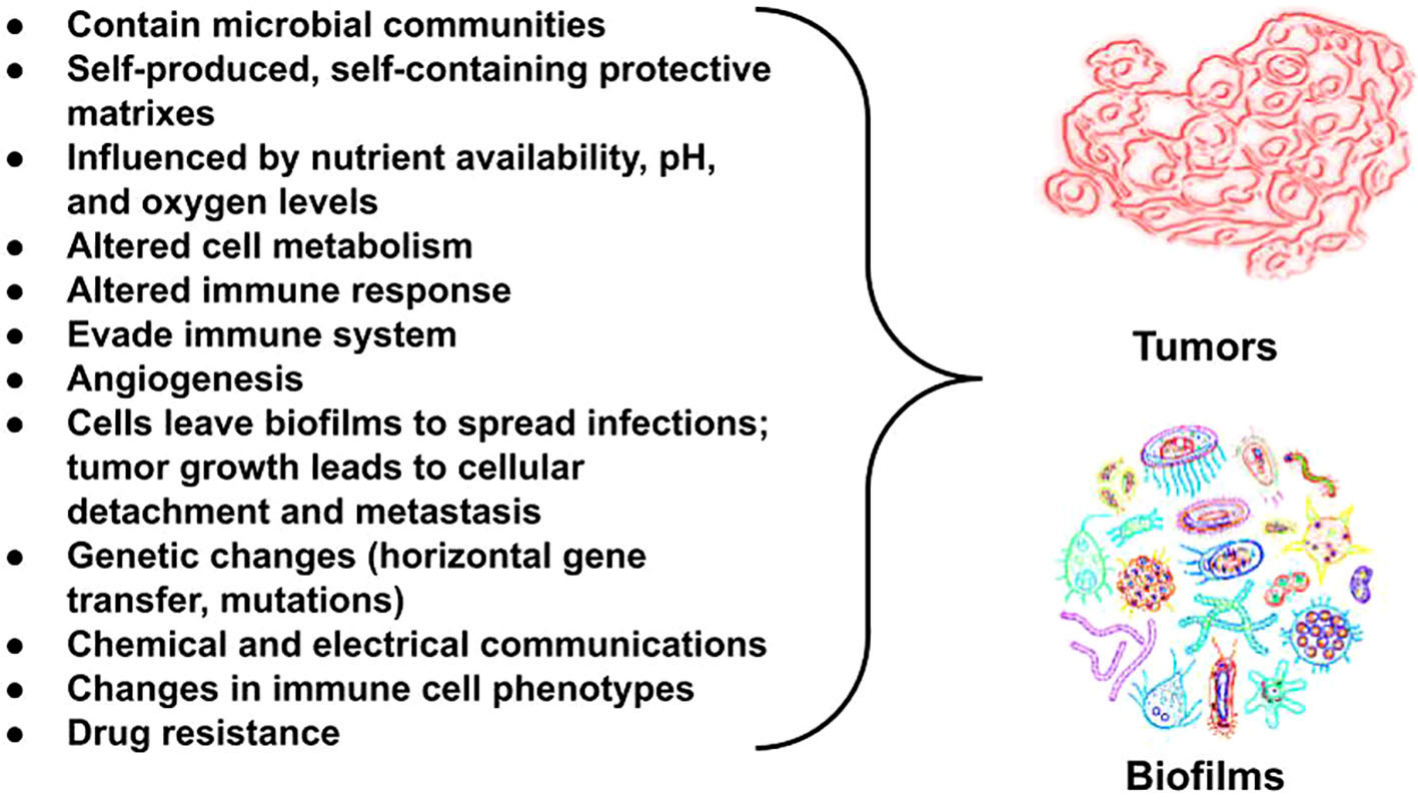
Bacterial biofilms and tumors share commonalities in their microenvironments, particularly in how they are influenced by the availability of oxygenation, micronutrients, pH levels, and the presence of bacterial metabolites. Both create outer matrixes that protect the cells within and make treatment challenging. Importantly, metabolic changes are seen in both. Metabolic changes occur frequently in both host cells and pathogens across biofilm-associated diseases. Metabolic reprogramming is a hallmark of cancer (reviewed in Mirzaei et al. (79)). Tumors and metastasis trigger melanogenesis via changes in pH. The extracellular space among tumor cells compared with normal cells have a pH difference of one unit (80). An increase in extracellular pH from 5 to 6.8 triggers maturation of melanosomes, which is where melanin pigments are synthesized (81). We theorize melanin helps fuel cancer.

Consider the microbiome to be the forest. Forests are connected by communications through fungi, and, in the case of a microbial biofilm, and chemical and electrical signaling (as we discuss later). Biofilms include a variety of microorganisms, including bacteria, viruses, fungi, protozoa, and archaea (70) as well as algae and small protists (71). Their populations vary by location and form various microbiomes, which may be commensal, symbiotic, or pathogenic (71). In nature, bacteria are rarely found in planktonic form. Rather, they mostly live in biofilm communities (72), where they adhere, proliferate, form micro communities, and secrete extracellular polymeric substances (EPSs) (73), which form the structure of biofilm matrices (74). Human cells/tissues, too, are connected by an extracellular matrix (ECM) (75). Hence, cells/tissues, biofilms, and tumors (76) all have extracellular matrices. In normal tissues, the ECM ensures tissue homeostasis and proper functioning. It provides structural support and biochemical cues for resident cells and is composed of proteins and other molecules (77) with various biochemical properties that regulate cell growth, differentiation, motility, and survival. Loss of ECM homeostasis is a hallmark of cancer (78). Of importance, there are many similarities between biofilm matrices and tumor matrices. (**Figure 2**). The main structural components of the tumor matrix are collagens (for the significance of collagen, see section 22, "Tumor cells and TAMS scavenge cysteine from the ECM for pheomelanogenesis"), which are synthesized in fibroblasts. Hyaluronan, or hyaluronic acid, (HA) is also an important component of the matrix, both in vertebrates and in microbes (82–85). Its antimicrobial effects will be discussed later.

## 5 Biofilms may be key to tumors/metastasis

Pathogens are often studied as singular, independent organisms, despite the fact that *in vivo*, they most often live in multispecies biofilm communities (86). Biofilms are three-dimensional structures made by complex communities of (predominantly) bacteria encased in a protective matrix (87). The biofilm matrix may be slime or plaque, the latter of which can become hard. We theorize these could describe blood cancers and solid tumors, respectively. The biofilm is important to virulence by providing physical resistance to antimicrobials and a way to hide from the host’s immune system (88, 89). Close proximity of the microbes may allow for the transfer of resistance genes (90) and viral recombination (86). Research suggests that bacteria are capable of purposefully leaving a biofilm, presumably to spread and form new biofilms. Also seen in tumors and biofilms is the breakaway of cells when the biofilm nears a critical thickness at which point it releases planktonic bacteria to colonize new surfaces (73). There is evidence that quorum sensing (see below) controls dispersal (reviewed in Parsek and Greenberg (91)).

## 6 Chemical and electrical signaling/properties in biofilms and tumors

The microorganisms in biofilms communicate both through chemical quorum sensing (87) and electrical signaling. Bacteria can communicate amongst themselves within the biofilm, with bacteria that are outside of the biofilm to recruit them (92), and with bacteria in other biofilms for mutual survival (92), independent of species, as the electrical signaling is generic (93). Bacteria in distant biofilms use electrical signaling to share nutrients when supplies are low in a coordinated strategy that enables the biofilms to increase their growth (55). Inside the biofilm, interior cells send electric signals through a ripple of cell-to-cell communications to the exterior cells when their glutamate reserves are depleted, causing the peripheral bacteria to stop dividing and the biofilm to stop expanding until more glutamate is available (94–96). The electric ripple seen in cell communication and biofilms appears very similar to the rapid fluctuations in electrical activity seen amongst breast cancer cells (97). Moreover, cancer cells can be differentiated from normal cells in the same tissue by their electrical properties, including frequency (98) as can infected cells (99–102) and (similar to pathogens) use electrical signaling (103). Further, electrical changes are observed in cancer cells that are metastasizing (97). Importantly, researchers have also demonstrated that small amounts of electricity can be used for gene expression; therefore, bacteria can control genes (104, 105). Similar to bacterial biofilms, viruses also form biofilms or colonize pre-existing biofilms (86). Electrical signaling, we postulate, is one way microorganisms control tumor growth and metastasis.

## 7 Evidence of metabolic reprogramming of cells by pathogens

Viral and intracellular bacterial pathogens (IBPs) reprogram host cell metabolism in order to be able to replicate and live within the host cell. Both viruses and bacterial pathogens use phagocytic immune cells, especially monocytes and macrophages, as well as dendritic cells, as hosts, in addition to non-professional phagocytes (epithelial cells, fibroblasts, and endothelial cells). The metabolism of these immune cells does not meet the nutrient requirements for the pathogens to replicate, especially when infected with IBPs, which, unlike viruses, must rely on their own biosynthesis machinery and ATP to sustain themselves and to replicate. In order to do this, both viruses and IBPs highjack and reprogram the metabolism of the host cell to meet the nutrient, energy, and metabolite requirements of the hijacking pathogen. There is evidence suggesting the strategy involves interactions with oncogenes and tumor suppressors, or the introduction of virus-specific oncogenes, central metabolic regulators. In some instances, the IBP is released into the cytosol of the host cell after the primary phagosome has been lysed, where it reprograms the host cell’s metabolism and adapts its metabolism to that of the host cell (reviewed in Eisenreich (106)). This is a key piece of evidence suggesting pathogen capabilities and reprogramming of immune cells may be more extensive than previously thought.

## 8 Phagocytes as Trojan horses in cancer

**Figure 3:**
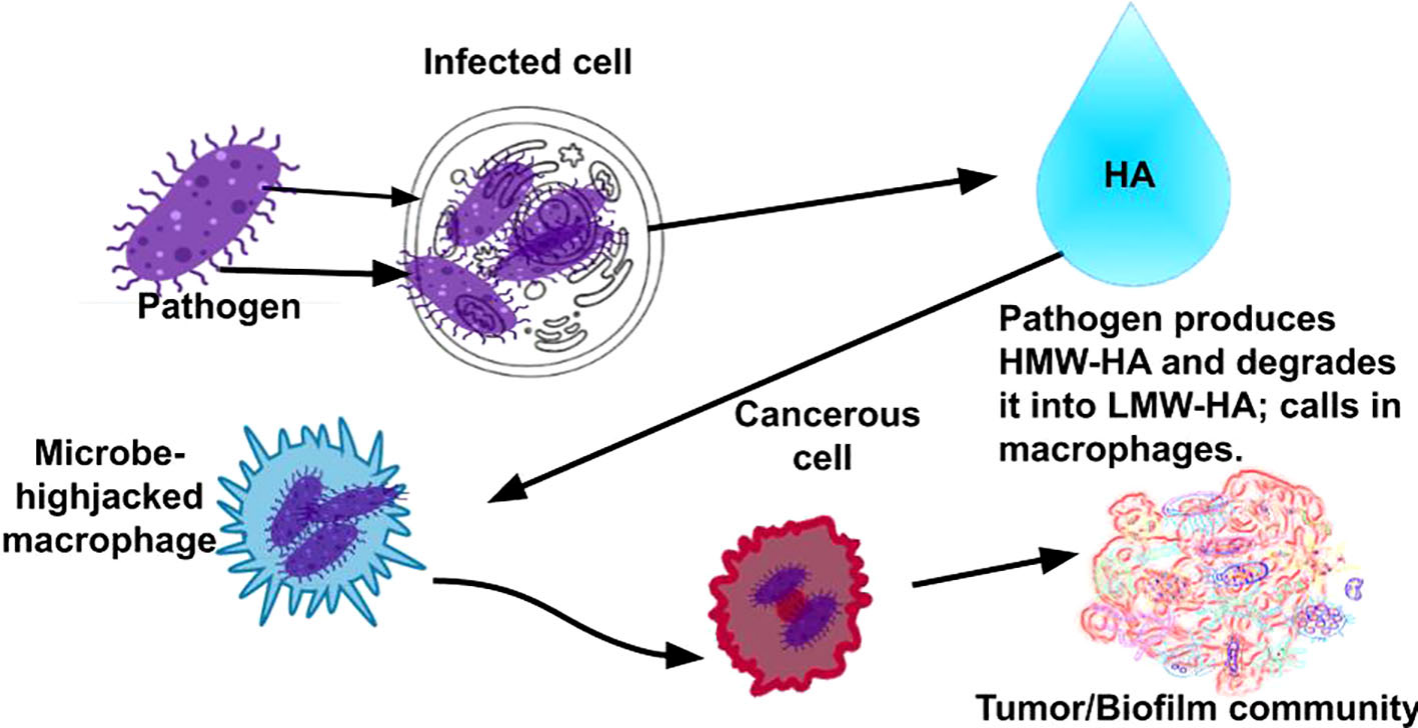
Rather than cancer cells slipping *by* the immune system, as conventionally believed in cancer, we provide evidence that pathogens slip *int*o the immune system via phagocytic immune cells, which allows the pathogens to hide from the immune system, infect other cells, proliferate, form complex microbial communities (tumors), and, eventually, become invasive cancers.

The next question to ask would be how does cancer spread undetected by the immune system? The conventional belief is that cancer cells are not differentiated enough from healthy cells that the immune system can detect them. However, we offer a different explanation: Rather than cancer cells slipping *by* the immune system, pathogens, which we theorize create cancer cells, slip *into* the immune system. Monocytes are phagocytic immune cells. Phagocytes engulf and kill pathogens. Monocytes differentiate into macrophages and dendritic cells. As immune cells, their roles are phagocytosis, antigen presentation, and cytokine production, and despite that they are immune cells, they are, interestingly, associated with tumorigenesis. Systemic and local microenvironmental changes triggered by the tumor “influence the phenotype, differentiation, and distribution of monocytes” (107). That is, monocytes and their related cell subsets regulate tumor growth and metastasis. In fact, monocytes and their derived cells, TAMs, which are also phagocytes, are found in the tumor microenvironment. There are two polarized forms of macrophages. M1 macrophages are anti-tumor and M2 macrophages are predominantly pro-tumor. TAMs are predominantly M2-like macrophages (108). TAMs play a role in tumor development, angiogenesis, metastasis, drug resistance, and immune system suppression (107, 109). Macrophages have been shown to infiltrate tumors, and increased numbers of macrophages present in tumors are associated with a poor prognosis. Tumor cells can attract macrophages to promote their survival and stimulate tumor angiogenesis (110). Tumor cells remodel phagocytes to promote tumorigenesis by increasing their numbers and affecting phenotype (107). (**Figure 3**) The question then arises, how are they able to do this? Some pathogens that infect macrophages intracellularly are able to change the macrophage polarization from M1 to M2 in order to support their survival and proliferation (111). The next logical question would be why would an immune cell work in favor of a tumor? We theorize that the reason that M2-like macrophages are seen in tumors in great numbers is because pathogens are hijacking the macrophages and reprogramming the macrophages to polarize to the M2 phenotype in order to promote their own survival, replication, and proliferation and to serve in metastasis, discussed in the next section. While monocytes mainly differentiate into macrophages in the tumor environment, some monocytes differentiate into dendritic cells. Dendritic cells typically are anti-tumor immune cells that are able to summon cytotoxic T-cells, which kill tumor cells. However, when dendritic cells present tumor-associated antigens on their surface in order to summon cytotoxic T-cells to destroy the tumor, TAMs will degrade those tumor-associated antigens to protect the tumor (107). This is further evidence that TAMs are hijacked by pathogens to serve as the tumor “bodyguards” in order to promote the intracellular pathogens’ survival and proliferation. We theorize that the pathogens are hijacking these phagocytic immune cells, after being engulfed by them, to release the factors that are known to cause monocytes to differentiate into M2-like TAMs (107) (**Table 1**). (Note: While the M1-M2 paradigm does not fully capture the complexity of macrophage behavior *in vivo*, we use it here to provide a useful framework for understanding macrophage polarization, as it references earlier research that is useful here.)

**Table 1:** Phagocytes house and transport cancer-inducing pathogens.

Type of Immune Cell	Phagocyte?	Effects on cancers	References
Macrophage	Yes Professional phagocyte	Protumor Metastatic progression, cancer-associated inflammation, helps cancer cells to resist therapy, involved in every step of cancer progression, angiogenesis, lymphangiogenesis, cancer cell proliferation, epithelial–mesenchymal transition, suppressing anti-tumor immune cells	(112, 113)
Natural killer cell	No	Antitumor Kills tumor cells and triggers apoptotic pathways in tumor cells	(112)
Neutrophil	Yes Professional phagocyte	Protumor Inflammation, tumor cell proliferation and invasion, tumor growth, angiogenesis, suppression of T cells, helps disseminated cancer cells survive and proliferate	(112, 113)
T cell	No	Generally anti-tumor Kills tumor cells.	(112)
B cell	Yes	Protumor*	(112, 114)
Dendritic cell	Yes Professional phagocyte	Protumor and Antitumor** Primes T cells.	(112, 113)

**Table 1** Phagocytes are tumorigenic. In examining the evidence for our theory, we made a remarkable discovery – all immune cell types that do not phagocytize are antitumor and the presence of increased numbers of these cells in the tumor environment is associated with a favorable prognosis, and immune cell types that do phagocytize, with the exception of dendritic cells**, are protumor and the presence of increased numbers of these cells in the tumor environment is associated with a poor prognosis (112). We theorize that microbes that can cause cancer, when engulfed by phagocytes, hijack their controls and use them as Trojan horses to infect healthy cells, escape eradication, and travel to new sites unnoticed (metastasize). In doing so, they are able to evade not only further immune system detection but cancer treatments, including stem cell transplants. *While B cells can phagocytize pathogens (114), we theorize that the main reason B cells have pro-tumor effects is because plasma cells (effector B cells) secrete antibodies that bind to pathogens, which tag them for phagocytosis by macrophages and neutrophils (115). Thus, B cells help the cancer-causing pathogens to get phagocytized, which then allows the pathogens to hijack the phagocytizing cells. **Dendritic cells are professional phagocytes; however, the microenvironment inside of the phagosomes of dendritic cells is less extreme than the microenvironments inside of the phagosomes of macrophages and neutrophils (113). We theorize that the reason dendritic cells have anti-tumor effects, despite that they are professional phagocytes, is because the less destructive microenvironment of the dendritic cells’ phagosomes is not suitable for the pathogens, as these pathogens have evolved to survive in more extreme microenvironments akin to the phagosomes of macrophages and neutrophils. Despite that, tumor-infiltrating dendritic cells can switch roles from immunostimulatory to immunosuppressant as tumors progress and become metastatic (116) via interaction with TAMs (116, 117), which we theorize is through microbial control, either directly from the pathogens in the macrophages or from pathogen transfer from the macrophage into the dendritic cell. These immunosuppressive dendritic cells are recruited by the tumor (117).

Our theory for the presence of pro-tumor immune cell roles explains the significant increases in the number of monocytes in cancer patients. The numbers of monocytes are especially high in individuals with cancer with histories of smoking and drinking (118), both of which we theorize provide enormous amounts of energy to the pathogens in the cells. In addition, we theorize that different communities of pathogens cause different cancers. Further, and intriguingly, it appears there is a correlation between an increase in inflammatory regulators and monocyte migration with increased expression of immune regulatory receptors and pro-angiogenic factors and that these monocytes that promote inflammation also “promote metastatic recurrence when systemic or local inflammation escalates under therapeutic interventions for primary tumors” (107), which suggests to us that the pathogens are responding to an attack by relocating. It is noteworthy that phagocytes, known for their role in engulfing and killing pathogens, also capture and process foreign particles. This includes carbon and various pigments (119). Additionally, melanin granules have been observed in lymphocytes during the inflammatory response, as well as in leukocytes, monocytes, and macrophages (120, 121). We theorize these melanin granules contribute to microbial growth and virulence similar to the way pigments produced by pathogens (122) or usurped by pathogens do (20) and promote tumor growth and metastasis. Finally, and remarkably, Lee and colleagues (123), for the first time, were able to capture and observe the behavior of macrophages and cancer cells. After injecting cancer cells into a mouse tail vein, they found the cancer cells travelled into the bone marrow. During the first hour, there was “serially sustained” contact between macrophages and cancer cells, and the cancer cells were engulfed by the macrophages. After 24 hours, this contact decreased significantly. We theorize that the healthy macrophages engulf the cancer cells to destroy them, and because the cancer cells are, according to our theory, infected cells, these pathogens are what are engulfed by the macrophages, and these pathogens reprogram the macrophages from M1 to M2 to not destroy the tumor community, and, hence, they retreat. In the future it would be interesting to follow the macrophages to see if they further spread the cancer and under what conditions. Kim (124) notes instances of pathogen use of Trojan horse mechanisms to cross the blood brain barrier using phagocytes. We would expect in these cases the spread of cancer to the brain, which we suggest cancer cells often migrate to because of the abundance of energy found there, in large part due to the presence of neuromelanin (20).

Roh-Johnson and colleagues (125) found through live imaging that tumor cells recruited macrophages, which shared cytoplasm with the tumor cells, after which the tumor cells broke off and metastasized; we see as further evidence that the macrophages were hijacked by already hijacked tumor cells. The group provided further details on the interactions between macrophages and tumor cells from their work and that of other researchers, noting that macrophages and tumor cells communicate with each other through various means, which makes sense from our perspective of a biofilm communicating with hijacked immune cells.

This leads to the question of what happens during the hijacking? Yang et al. (126) investigated TAMs and their interactions with breast cancer cells that lead to metastasis and found that macrophages regulate breast cancer cell invasiveness via oncogenic microRNAs (miRNAs) delivered to the cell by exosomes. miRNAs regulate an estimated 60 percent of human protein-coding genes (127). Viruses frequently take control of the miRNA pathway by depleting host miRNAs or by making their own miRNAs (128, 129). Pathogens exploit the host’s miRNA system for survival, replication, and pathogenesis within host cells and to evade certain immune defenses. miRNAs play an integral role in cellular development, differentiation, proliferation, and apoptosis and can control the host immune response and antibody production. miRNAs can affect activation of B cells, monocytes and macrophages, polarization of macrophages, and differentiation of monocytes (reviewed in Chandan et al. (130)). Some pathogens produce their own miRNAs within the host, which further supports their replication, survival, and/or latency (131). Bacterial and viral miRNA (specifically) increase bacterial and viral proliferation, increase virulence, and manipulate the host responses to provide an improved environment for the pathogens (reviewed in Nosanchuk and Casadevall (122)). This sheds further light on how pathogens can build tumors and metastasize. (Of note, it is known that there is a bidirectional regulation of p53 and miRNA.)

We believe the significance of our discoveries of the linkages between complex biofilms, phagocytes, melanin, platelets (discussed later) and tumorigenesis cannot be overemphasized.

## 9 The intricacies of metastasis suggest pathogen orchestration

**Figure 4:**
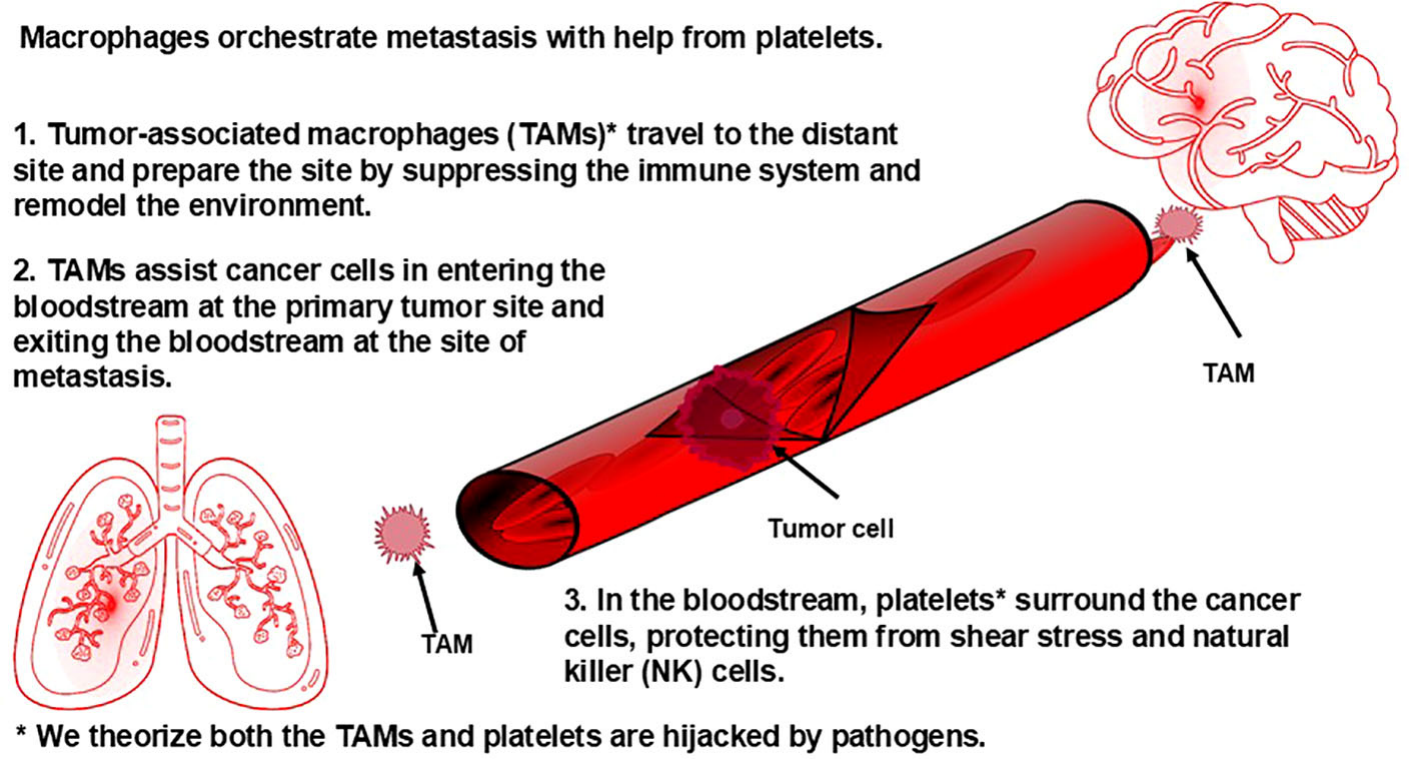
Macrophages orchestrate metastasis with help from platelets.

The orchestrated roles that platelets and macrophages play, along with other immune cells, in metastasis begins with TAMs, which we theorize are genetically programmed by pathogens. It has been observed that TAMs from primary tumors travel to the distant site of future metastasis in advance of primary tumor cells and prepare the site for these cells through secretion of various enzymes, including those enzymes that induce extravasation, the movement of cells out of a blood vessel into tissue during metastasis, and angiogenesis. Tumors use angiogenesis to support proliferation (132) and metastasis (133). (Angiogenesis also occurs in various bacterial, viral, protozoan, and fungal infections. Angiogenesis caused by pathogens can be categorized into two types: one where the pathogens directly trigger the host to form new blood vessels through the pathogen’s own molecules, or the formation of the vasculature is a result of a general inflammatory response from the host (134)). At the same time, these TAMs also suppress anti-tumor activity of immune cells, such as dendritic cells and T helper 1 cells; cause the tissue-resident macrophages to become hijacked (which we theorize is due to pathogens spreading from the TAMs to the tissue-resident macrophages) and aid in preparing the microenvironment to support the arrival of the tumor cells; secrete molecules that force circulating tumor cells to the site they have prepared (135); and aid tumor cells in getting into (136) and out of (137) blood vessels (both of which have been captured on video (136, 137)), which involves staying in close contact with the tumor cells and helping the tumor cells survive while in circulation (reviewed in Lin et al. (135)). Traveling through the bloodstream presents certain challenges for the primary tumor cells, including surviving sheer stress from blood flow and evading detection by NK cells in the bloodstream. One strategy that circulating tumor cells use to overcome these challenges is sending exosomes containing mRNA and proteins into the bloodstream. The exosomes containing the mRNA and proteins are taken up by platelets. Platelets, it turns out, play a key role in metastasis: It is generally understood that the bloodstream is a primary route for many cancers to metastasize to distant organs; it is known that there is a significantly increased risk of thrombosis in individuals with cancer (138), with the risk of venous thromboembolism increasing as the stage of cancer increases (139); and it is known that tumor cells increase substantially the production of platelets through secretion of various molecules (140). The mRNA, we theorize, reprograms the platelets, which are described by other researchers as having become “tumor educated” (protumor). Remarkably, the circulating tumor cells are able to change how RNA is spliced inside the platelets, which, we theorize, contributes to the reprogramming of the platelets. These pro-tumor platelets adhere to the circulating tumor cells and then shield the tumor from the sheer stress of the bloodstream and protect circulating tumor cells from NK cells via secretion of TGFβ (transforming growth factor beta), a molecule that suppresses NK cells. (**Figure 4**) The circulating tumor cells also take specific membrane proteins from the platelets and incorporate them into their own membrane. These membrane proteins cause NK cells to fail to recognize those tumor cells as targets for elimination (reviewed in Heeke et al. (141) and Li et al. (140)). It is our view that cancer cells taking membrane proteins from platelets to disguise as healthy self-cells is a strategy that would be used by a pathogen or a self-cell under pathogenic control. In fact, pathogens do utilize platelets to avoid detection by NK cells in bloodstream infections. During sepsis, pathogens interact with platelets, leading to their activation and aggregation (142).

The precisely orchestrated interactions in metastasis we describe here provide further support for our theory that pathogens are at the helm in cancer, rather than cancer being a self-cell aberration. Further, the somatic mutation theory states that cancer arises due to chance mutations. However, the probability that a self-cell happens, by pure chance, to obtain a series of mutations that causes it to send out exosomes with the exact RNA sequences and the exact proteins necessary to cause platelets to be reprogrammed in such a way that allows the tumor cells to get through the bloodstream to another site in the body, all while taking specific membrane proteins from the platelets that allow the tumor cells to evade NK cells is too small to be realistic, especially considering the frequency of the event across hosts. And, while the probability of a particular point mutation occurring is extremely small, as we discussed in section 2, the probability of a series of mutations to cause such a precise event is infinitesimally smaller. Hence, we believe that the aforementioned tumor cell-platelet interactions are not only evidence against the somatic mutation theory, but further support our theory that pathogens are at the helm in cancer.

It is worth briefly considering here the similarities of metastasis to the spread of infection. Most cancers metastasize through the bloodstream, some spreading first via the lymphatic system (breast, lung, gastrointestinal cancers, all of which have been related to pathogens (143–145)) before entering the bloodstream. Certain other cancers spread locally via transcoelomic spread, for example, ovarian cancer can spread across the peritoneal cavity to the surface of the liver or other abdominal organs by shedding cancer cells (146) and other modes. (Ovarian cancer also has been linked with pathogens (147, 148).) These strategies for disease spread strongly resemble sepsis and local spread of infection, respectively.

## 10 Similarities of angiogenesis in infection and cancer

As we noted previously, bacteria can induce angiogenesis through various mechanisms, including the bacterial component lipopolysaccharides (LPS) (134, 149). LPS induces metastatic growth, which is associated not only with angiogenesis but vascular permeability and tumor cell invasion (149), as well. The presence of bacteria causes an inflammatory response, which triggers release of pro-angiogenic factors, including vascular endothelial growth factor (VEGF), fibroblast growth factor (FGF), and cytokines (134, 150). Similar to tumors, which have areas of hypoxia, infected tissues can have areas of hypoxia (151). This further stimulates angiogenesis as part of tissue repair, which uses the same key genes and signaling pathways used in tumor growth, including VEGF, FGF, and platelet-derived growth factor (PDGF) (152, 153). Angiogenesis helps in the migration of immune cells to the site of infection to aide in the clearance of pathogens (134), and it is certain of these immune cells that, according to our theory, get hijacked by the pathogens in cancer, as we discussed above. It is known that, similar to metastasis, pathogens exploit angiogenesis to enhance their survival and spread (134, 150). It is important to note that the host does not need to be infected in order for bacteria to participate in angiogenesis. Commensal bacteria are involved in angiogenesis under various conditions, for example, in wound healing, commensal bacteria in the skin and gut can promote angiogenesis by triggering the production of VEGF and other growth factors crucial for new blood vessel formation (150). Commensal bacteria also can be involved in angiogenesis in cancer (154).

Now that we have established that bacteria, in various ways, are involved in angiogenesis, a process without which tumorigenesis and metastasis could not exist, we underscore here something we believe is of great importance: The vasculature in bacteria-induced angiogenesis appears disorganized, irregular, and unevenly spread in looping form, all of which is also seen in malignant tumors (134, 150). Within the chaotic structure there is also a lack of conventional hierarchy of blood vessels in tumors (155) as well as in infected tissue (134). This can be compared with the vasculature in healthy cells, which is orderly in branch-like patterns (155). (Images can be viewed in (155)). In addition, there is increased permeability in the vasculature resulting in bacterial angiogenesis (134), which we discussed previously in this article in tumors and its role in metastasis (134). We theorize the areas of hypoxia and areas of dense vasculature within infected tissue and tumors are the result of different microbial communities with different abilities in terms of angiogenesis. Indeed, some bacteria are more adept at inducing angiogenesis than others, and some bacteria are also capable of inhibiting angiogenesis, resulting in hypoxia (reviewed in Sajib et al. (150)). We theorize areas of angiogenesis versus areas of hypoxia in tumors may be the result of turf wars, where areas of increased vascularization appear to be areas of more successful pathogens in what appears to be competition for territory, nutrition, and oxygen.

## 11 Mechanisms for bacterial reprogramming of host cells

We assert throughout this paper that pathogens are reprogramming cells. While pathogens can mutate host genes, they have additional sophisticated mechanisms to manipulate host cell processes, which is important, because genetic mutations do not fully explain tumorigenesis and metastasis. We focus here briefly only on bacteria to present just a few of the ways in which microorganisms can manipulate host cell processes by hijacking host cell machinery (156). *Listeria monocytogenes* is an interesting example, because it first infects the macrophages that engulf it. It then multiplies in the cytoplasm, where it uses the protein ActA to hijack the host cell’s actin polymerization machinery to form a tail of actin filaments. It uses the tail to push itself against the host cell membrane and create protrusions into adjacent cells, where the bacterium is engulfed and repeats the cycle, spreading infection into more host cells (157). In tumors, actin is known to play important roles, including roles in gene expression and transcription, metastatic migration, survival in the bloodstream by protecting the tumor cells from being degraded and aiding in attachment to platelets, and extravasation (158), and intracellular pathogens are known to be able to hijack polymerization of actin and hijack actin-associated proteins in order to rearrange actin structures for their benefit (159). Furthermore, actin and actin-associated proteins are known to accumulate in the nucleus in many tumor cells, which is important to note when considering that actin can affect gene expression, and intracellular pathogens can affect actin structuring (158). Another form of cellular programming is seen in intracellular infection with *Mycobacterium leprae*, which revert host cells to stem cells/progenitor cells by manipulating the host cell signaling pathways and epigenetics, including histone modifications (160) and DNA methylation (161). *M. leprae* can also spread infection via macrophages (160, 162).

While the entirety of the sophisticated strategies used by microorganisms to manipulate host cell processes is too numerous to detail here, it is important to note that mechanisms used by microorganisms are commonly observed in cancers that have not be identified to have an infectious etiology, which is the vast majority of cancers. These include evading the immune system through expressing proteins that inhibit immune cell activity or creating an immunosuppressive microenvironment (163); manipulating host signaling pathways that control cell growth (164); triggering genomic instability (165); inducing metabolic reprogramming (164) and involving chronic inflammation, a hallmark of both pathogens and cancer (166).

Research demonstrates tumor-related microbes regulate oncogenic signaling pathways; modulate immune responses; and tumor microbiota affect drug efficacy/metabolism. In addition, microbiota-derived metabolites play a role in tumor progression (167).

## 12 The relationship between cancer and inflammation: A new understanding

Conventionally, cancer is thought to result from and progress due to inflammation (168). Yet, not all chronic inflammation leads to cancer. We theorize that inflammation associated with cancer is the result of the inflammatory response to infection. Professional phagocytes, specifically neutrophils and macrophages, organize the triggering and resolution of inflammatory responses. Once pathogens are captured, they are encapsulated in an intracellular vacuole where, after maturation, killing mechanisms are triggered. They are both highly migratory cells, and they are not always successful for a variety of reasons (reviewed in Linnerz and Hall (169)). Inflammatory pathway activation, which promotes the elimination of pathogens and inhibits their growth, typically follows phagocytosis (170). De Visser et al. (171) found that B lymphocytes, another type of immune cell, are required for the chronic inflammatory states associated with cancer. They found eliminating these cells in mouse models prevents chronic inflammation and angiogenic vasculature formation, whereas their presence reinstated these conditions, which are required for malignancy. Effector B cells produce antibodies, which bind to pathogens. In doing so, B cells tag these pathogens for phagocytosis, which triggers cancer-causing pathogens to be phagocytosed by macrophages and neutrophils. Being phagocytosed allows the pathogens intercellular access wherein, we theorize, they hijack the phagocytosing cells and cause/spread cancer. Using *Francisella tularensis*, a cytosolic IBP, researchers showed the bacteria replicates in the cytosol of macrophages. Uninfected macrophages acquire *F. tularensis* from contact with infected macrophages. Other researchers have demonstrated that *Salmonella enterica*, which can live in host cell vacuoles, are transferred among macrophages in the same way (reviewed in Bourdonnay and Henry (172)). It is these infected macrophages, similar to monocytes, that we believe are key to understanding tumor growth and metastasis. Interestingly, B cells also can be phagocytic (173).

## 13 Cellular hijacking/metabolic changes can explain increased glucose

Cancer cells are recognized to have upregulated glycolysis, resulting in increased glucose consumption (174). Pathogens utilize glucose in infected cells. To replicate efficiently within host cells, cytosolic IBPs use a dual-part metabolic process that relies on glycerol, pyruvate, and cysteine (the latter of which we theorize is critically important in energy, as it is a precursor to pheomelanin), along with, potentially, other amino acids, for energy (reviewed in Eisenreich (106)). (Indeed, many mutations in cancer cells result in cysteine being substituted for other amino acids. Research suggests that cancer cells use cysteine to overcome the challenges of dwindling metabolic substrates and rising reactive chemical species resulting from high energy use in rapid proliferation (reviewed in Nin et al. (175)). The IBPs in vacuoles also use a dual-part metabolic process, although using different pathways. Further, similar to the cytosolic IBPs, most vacuolar IBPs have the genes necessary to convert pyruvate to acetate to generate ATP (106).

Further supporting our theory, it has been observed that certain immune cells, notably lymphocytes such as CD4+ T cells, B cells, and M2 macrophages (M2-MPs) that are alternatively activated, exhibit a metabolic state that is conducive to the propagation of various viruses. For example, the human immunodeficiency virus replicates effectively in CD4+ T cells, and the Epstein-Barr virus is known to replicate in B cells. Similarly, certain IBPs, including *Salmonella* and *Brucella*, have been found to replicate within M2-MPs (reviewed in Eisenreich (106)). All of these pathogens have been linked to the development of cancer (176–179).

As we discussed previously, live bacteria have been found in macrophages/monocytes. Further, the changes in cell metabolism that occur during infection are also seen in cancer cells. IBPs have what appears to us to be a commensal relationship with the host cell by using host-derived carbon compounds that are less critical for the host’s own energy supply. (It is noteworthy that all risk factors we could find, with the exception of infection, are sources of carbon, as discussed later in this article.) These carbon compounds include mainly pyruvate or a metabolite that can be converted into pyruvate – cysteine, lactate, glycerol, or serine. Pyruvate is a pivotal molecule in metabolism. During glycolysis, host cells can break down glucose through a series of enzymatic reactions to produce pyruvate (reviewed in Eisenreich (106)). We theorize that the increase in glycolysis observed in cancer cells (174) is due to IBPs using pyruvate to generate their own energy supply. Alternatively, IBPs can transform pyruvate into glucose via gluconeogenesis for building their unique membrane surface structures. These structures are specific to IBPs and cannot be synthesized by the host cell’s pathways (reviewed in Eisenreich (106)). (We view using pyruvate versus glucose from the host cell as more efficient for the bacteria, because it allows them to skip the 10-step process of glycolysis. More often than not, these bacteria will need pyruvate to produce ATP for energy, rather than glucose for their membrane structures.) Hence, IBPs have evolved a sophisticated way to exploit the host cell’s metabolism to their advantage, ensuring their survival and replication while not completely depleting the host’s resources. The work by Eisenreich’s group also provides insight into the genetic basis for these processes, indicating that the necessary enzymes for gluconeogenesis are present in many IBPs, supporting our theory that they actively manipulate host cell metabolism for their own benefit. Additional parts of the bipartite metabolism strategy are described by Eisenreich et al. (106), who also note that some pathogens use fatty acids or cholesterol from the host cell for energy components. While the bipartite metabolism strategy permits intracellular bacterial replication, the expression of the virulence factors required for intracellular bacterial replication is often blocked when the major carbon source for IBPs is glucose (106). We presume the blockage is controlled by the host cell and deemed not necessary to change by the IBPs due to their workaround. From our perspective, it is also important to note that pyruvic acid/ethyl pyruvate inhibits melanogenesis in melanoma cells (180). This supports our theory that there is an inverse relationship between ATP production and melanogenesis (20), even in pathogens. Also of interest is a possible direct supply of melanin through immune system cells. Lymphocytes in the inflammatory response contain melanin granules. Wassermann found that in an inflammatory reaction, neutrophils collect small particles of melanin. These particles clump together to form larger particles, as the neutrophils diminish in size. These particles are transferred to lymphocytes through phagocytosis of neutrophils as well as other immunological methods. Wassermann notes these melanin-containing lymphocyte cells have been found intracellularly inside fibrocyte-like cell macrophages (120).

As we noted previously, viruses control the host cell’s catabolic and anabolic pathways, mostly through oncogenes and tumor suppressors, which may lead to controlling metabolic pathways. Much less is known about IBPs, but they also have been shown to affect oncogenes and tumor suppressors in altering cell metabolism. IBPs oftentimes replicate in the same host cells as do viruses. Unlike viruses, IBPs have their own metabolism that they adapted to the metabolism of the host cell, and it is difficult for researchers to separate the two to measure them independently. However, the choice of cells in which to replicate and metabolic reprogramming of host cells by the virus could be supporting bacteria co-infection (reviewed by Eisenreich (106)). This leads us to an interesting question of whether tumor growth is made more efficient by viral setup of the host cell metabolism for bacterial replication and supports our theory that tumors are mixed colony biofilms.

Individual tumor cells have different metabolisms (181). An illuminating illustration of different IBPs having different metabolic needs and altering host cell metabolism to meet their (the IBP’s) metabolic needs is as follows: *Listeria monocytogenes* was shown to activate metabolic pathways related to energy production and cell growth in bone marrow derived macrophages (BMDMs) after infecting them, which allowed for their growth in the immune cell. In contrast, when *L. monocytogenes* infected J774 immortalized cancer cells, cell metabolism was downregulated. Reduction of the p53 protein increases bacterial growth, while overexpressing p53 inhibits it (reviewed in Eisenreich (106)). In cancer, p53 is inhibited. Eisenreich and colleagues noted an “apparent discrepancy between the metabolic host cell responses of primary and cancer cells upon infection by the same [IBP]” (106). Applying our theory provides illumination on their observation: J774 are tumor cells. *L. monocytogenes* increases the energy production of normal cells but lowers energy production of J774 tumor cells. The J774 tumor cells were likely created by a pathogen other than *L. monocytogenes*. Because *L. monocytogenes* did not make that cancer cell line, it had to alter the cell’s metabolism in order to replicate. These experiments provide further evidence that the bacteria can take over cells and alter their metabolism, and it further supports our theory that pathogens are transforming host cells into tumor cells. Riley and colleagues (36) found that bacteria integrate their DNA into the human genome, insertion is upregulated in tumors, and these insertions occur more frequently in the mitochondrial genome. This could help to explain changes in energy metabolism seen in tumor cells. Furthermore, these investigators found evidence that bacterial DNA insertions cause upregulation of transcription of four proto-oncogenes, converting them into oncogenes, in stomach adenocarcinomas.

## 14 IBPs, metabolic regulators, and cancer

**Figure 5:**
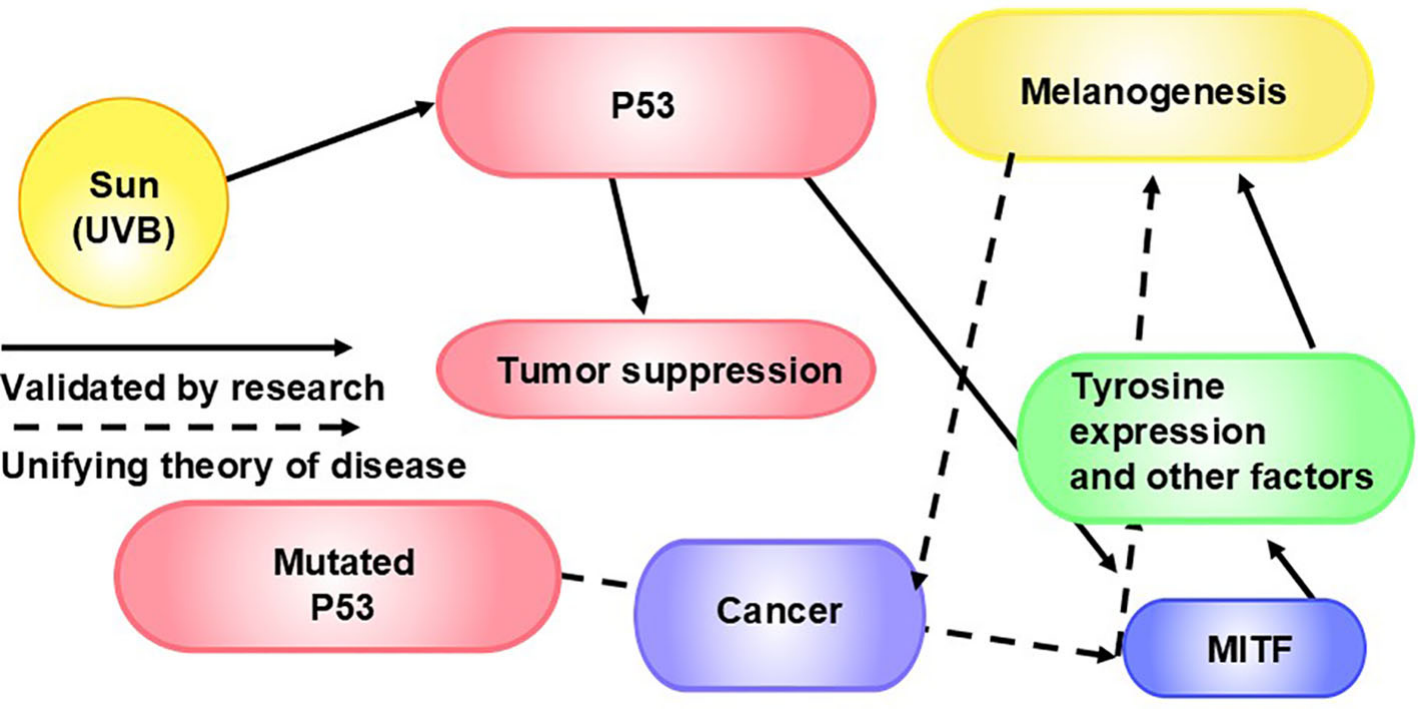
P53, MITF, and Melanogenesis in Cancer: Ultraviolet radiation induces melanogenesis via p53 (186), a key regulator of melanogenesis (185). P53 plays an important role in tumor suppression (187). The p53 mutation leads to about 50% of cancers (187, 188). P53 controls MITF and, consequently, tyrosinase expression (185). Tyrosinase is necessary for melanogenesis (189). MITF regulates the development and function of melanocytes (190). As such, MITF is a master regulator of melanogenesis. Not surprisingly from our point of view, MITF is also involved in tumorigenesis (191), we theorize via factors including melanogenesis.

Of key importance to our theory is the impact of IBPs on the central metabolic pathways of the host cell. Pathogens initiate the activation of certain parts of the PI3K/Akt/mTOR signaling pathway and the Myc oncogene (106). In cancer cells, the PI3K/Akt/mTOR pathway plays a crucial role in cell metabolism, growth, and motility and often results in increased uptake of glucose, heightened aerobic glycolysis (106), cell proliferation, autophagy, apoptosis, angiogenesis, and chemoresistance (182). (The PI3K/Akt/mTOR pathway is also inversely connected to the control of melanogenesis via melanocyte inducing transcription factor (MITF), a master regulator (183, 184), which is important to consider in the context of our theory on the fundamental role of melanin in cellular energy production (20). P53 can modulate MITF activity and tyrosinase expression, influencing melanogenesis (185). (**Figure 5**) Alternatively, IBPs may modify the levels and activity of p53, a tumor suppressor gene, and hypoxia-inducible factor 1 (HIF-1) (106). P53 is the most frequently mutated gene in human cancers. When functioning properly, P53 helps prevent tumor formation by responding to DNA damage by activating cell cycle arrest, so that DNA can be repaired, and/or triggering apoptosis (192). In response to hypoxic regions in solid tumors, HIF-1 induces the transcription of genes that regulate glucose metabolism, angiogenesis, cell proliferation, invasion, and metastasis, contributing to disease progression (193). There is an increase in uptake and use of glutamine in many tumors (194). We theorize that this, too, is due to IBPs, as some IBPs are able to cause increased uptake and use of glutamine via Myc and upregulate glutaminolysis (106). Also in support of our theory, glutaminolysis in macrophages causes polarization to the M2 macrophage phenotype (195), which is beneficial to the tumor. As further evidence that tumors are complex microbial communities, the PI3K/Akt/mTOR signaling pathway is also commonly overactivated in cancer (196), and alterations of P53 and HIF-1 are also commonly seen in cancer (17, 197). Activation of the Myc oncogene, too, is frequently seen in cancer, and inactivation of Myc results in tumor regression in many cancers through various mechanisms, which suggests to us inactivation of Myc cuts off the energy to fuel these mechanisms or simply cutting off fuel leads to proliferative arrest, senescence, apoptosis, interference with angiogenesis, and other mechanisms (reviewed in Felsher (198)). In our view, this is especially true in terms of “addiction,” where cancer cell survival is dependent on continuous activation of certain mutated oncogenes, which is further support of tumors being complex pathogen communities, as tumors typically recur after initially responding to therapies targeting oncogene inactivation (reviewed in Felsher (198)), just as pathogens mutate during and after insufficient antimicrobial treatments. We theorize that these upregulations and alterations in genes and pathways are triggered by IBPs within tumor communities.

Given that the PI3K-AKT pathway leads to the activation of mTOR, and the mTOR pathway is disrupted in cancer, it is interesting for us to discuss the drug Rapamycin in the context of these and other pathways, as it holds elucidations on our theory. Rapamycin inhibits mTOR complex 1 (mTORc1) and has both anti-inflammatory and anti-tumor activity as well as antibiotic effects. It is used to treat *Candida albicans*, which is frequently found in various cancers (48, 49) and frequently found in macrophages in cancers (50). ATP activates mTORC1. Adenosine monophosphate (AMP)-activated protein kinase (AMPK), an enzyme that plays a key role in cellular energy homeostasis, inhibits mTORC1 (199). Hence, AMPK is activated under conditions of low energy (high AMP/ATP ratio) and restores energy balance by inhibiting anabolic processes through phosphorylation and activating catabolic processes. This further supports our theory that ATP and melanin have an inverse relationship in providing energy to cells (20).

AMPK generally promotes catabolic pathways that produce ATP and at the same time inhibits anabolic pathways involved in different processes that consume ATP. As an energy sensor, AMPK is involved in the main cellular functions implicated in cell fate, such as cell growth and autophagy.

## 15 Dual effects of hyaluronan

**Figure 6:**
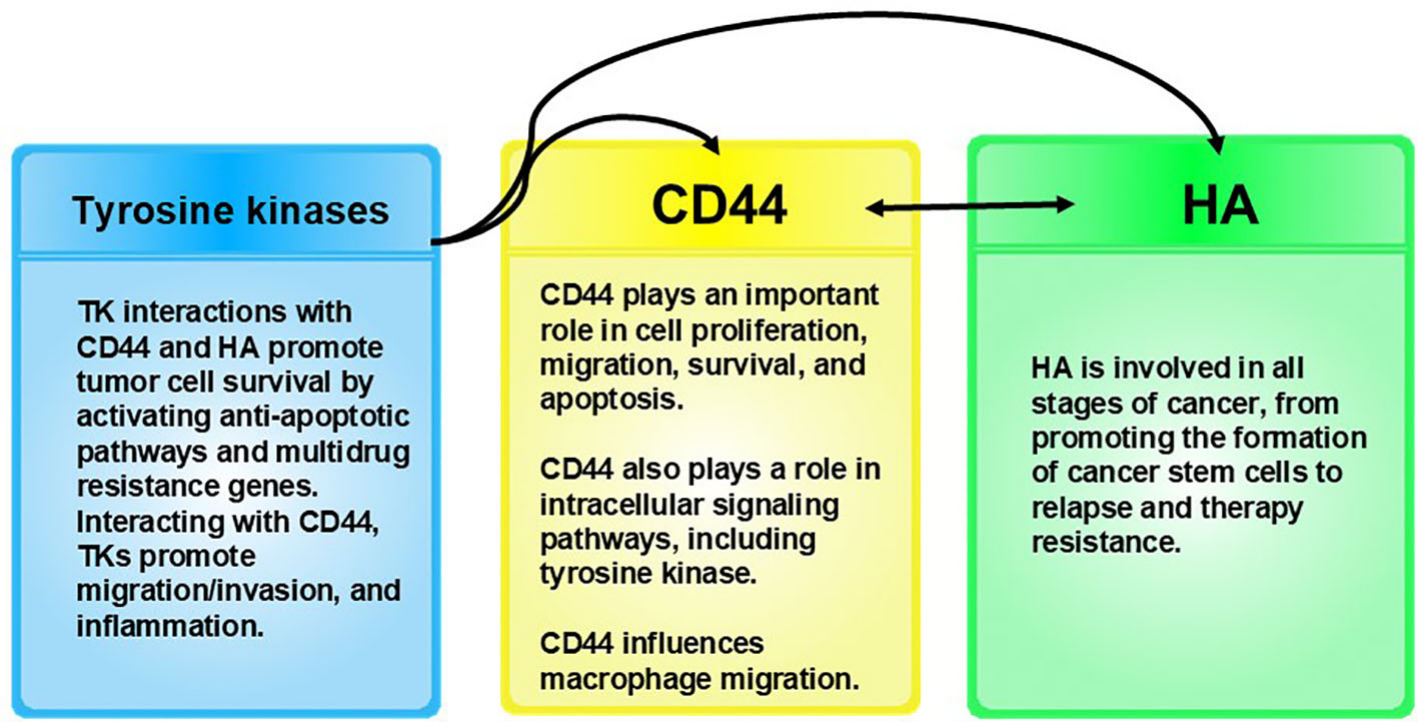
Receptor tyrosine kinases interact with CD44 and HA. These interactions promote tumor cell survival by activating anti-apoptotic pathways and multidrug resistance genes (202), and along with CD44, tyrosine kinases promote migration/invasion and inflammation. CD44 influences macrophage migration (203). It is important to note that expression of CD44 is elevated in tumor macrophages (204), which we theorize are highjacked by pathogens in cancer. CD44 also modulates signaling pathways involved in cancer cell proliferation, invasion, metastasis, and therapy resistance. CD44 expression levels can be used to indicate a poor prognosis in cancer (205, 206). CD44 appears to be involved with melanogenesis as a result of its interactions with HA, the main ligand for CD44 (205). HA binds to the CD44 ligand-binding domain, activating various signaling pathways that can influence melanocyte behavior, including proliferation, survival, and migration (205). The cKit receptor is a type of tyrosine kinase expressed on the surface of melanocytes. cKit receptor activation triggers intracellular signaling that can influence melanocytes, including proliferation, migration, and melanogenesis (207). While HA-CD44 interactions occur independently of the cKit receptor, both HA-CD44 interactions and cKit receptor activation are essential for melanocyte behavior, including melanogenesis, proliferation, and migration.

**Figure 7:**
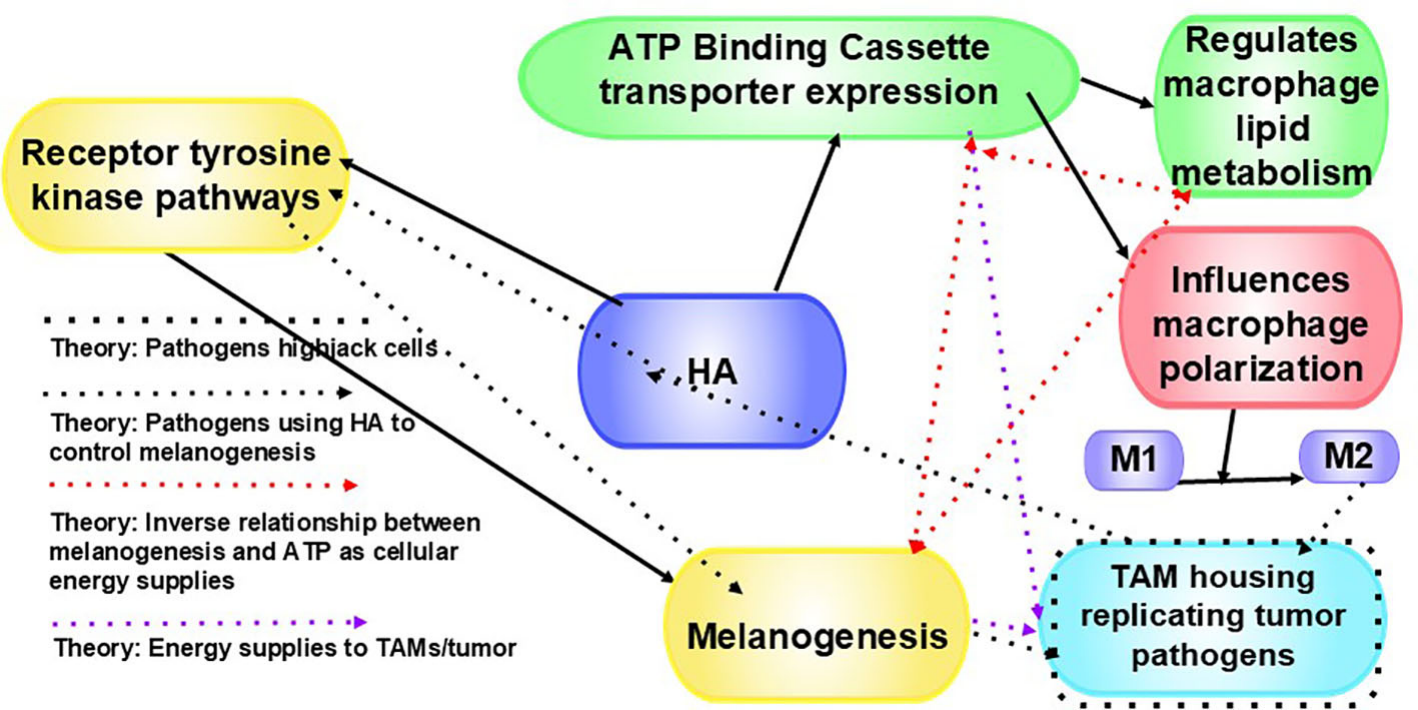
HA affects ATP-binding cassette (ABC) transporters. ABC transporters are a large family of proteins that utilize the energy from ATP hydrolysis to transport substrates (209). HA influences various mechanisms that regulate ABC transporter expression, transporters of which play a role in effluxing cancer therapies out of cells (208), affect lipid metabolism in macrophages (209), and influence macrophage polarization (210). HA also regulates receptor tyrosine kinase pathways, which influences cancer cell behavior. By affecting receptor tyrosine kinase pathways, HA influences certain cellular processes vital for cancer cells, including growth, motility, differentiation, and metabolism (208). This can lead to abnormal receptor tyrosine kinase activation, (208), which is common in many cancers. We theorize that pathogens highjack cells, pathogens use HA to control melanogenesis, there is an inverse relationship between melanogenesis and ATP as cellular energy supplies (20), and both melanin and ATP fuel TAMs and tumors.

**Figure 8:**
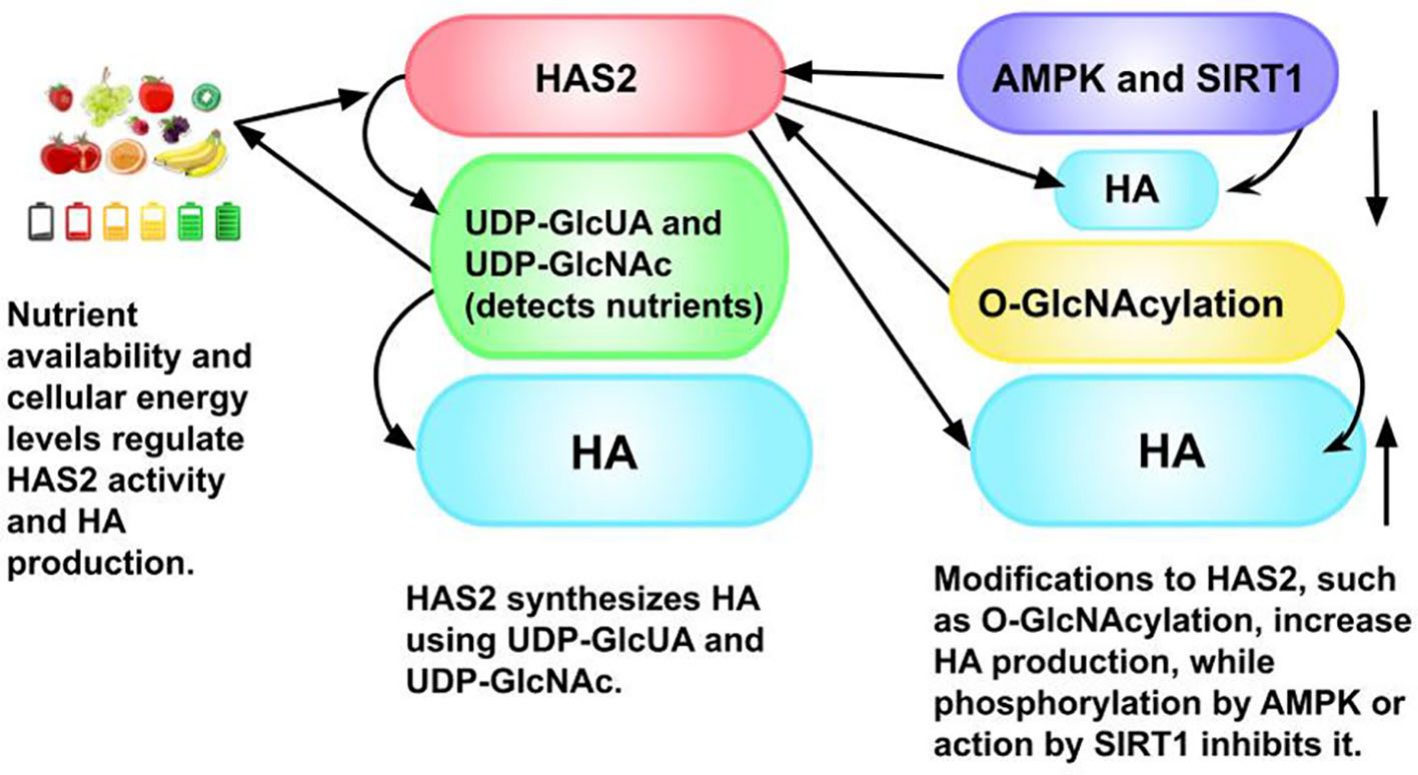
The process of hyaluronic acid (HA) synthesis and its regulation: There is an overproduction of both HA and HYAL in many tumor types. HA at specific molecular weights promotes angiogenesis and cell motility through the extracellular matrix to form metastases (216). Three HASes, but mainly HAS2, synthesize HA using as substrates uridine diphosphate (UDP)-glucuronic acid (UDP-GlcUA) and UDP-N-acetylglucosamine (UDP-GlcNAc), both sugars. UDP-GlcNAc both detects nutrients and is a donor substrate for the O-GlcNAcylation of HAS2, a cytosolic protein (215). (A donor substrate molecule provides a sugar group for a glycosylation reaction, which attaches a sugar to another molecule. This new sugar group is typically activated by a nucleotide, in this case, UDP, which results in a high-energy donor substrate. These substrates are used by enzymes, specifically, glycosyltransferases, that are involved in the biosynthesis of glycoproteins, glycolipids, and polysaccharides.) This post-translational modification increases the production of HA and stabilizes HAS2. HA secretion is inhibited when HAS2 is phosphorylated by adenosine monophosphate (AMP)-activated protein kinase (AMPK), an enzyme that plays a key role in cellular energy homeostasis, which is activated by low ATP/AMP ratios. ATP provides cellular energy, and AMP carries energy in cells (215). Sirtuin 1 (SIRT1), also an energy sensor, inhibits the expression of HAS2 and HA deposition in the pericellular coat (215), which is found around the cell. The pericellular matrix (pericellular coat) is found between the plasma membrane and the interstitial extracellular matrix (217). It is important to note both the involvement of HA and ATP as energy regulators and in homeostasis, as well as melanin or its precursor, phenylalanine, which plays a ubiquitous role in metabolic pathways, and, hence, is understood to exist in virtually all cells (218), as this is key to our theory that ATP and melanin have an inverse relationship in providing energy to cells (20).

HA is synthesized by fibroblasts, which make most of the extracellular matrix, and by keratinocytes and other cells (200). The process of HA synthesis and its regulation is important to our theory, because keratinocytes process and hold melanin, which is synthesized in melanocytes and transferred to keratinocytes by melanosomes (201). HA is involved in all stages of cancer, from promoting the formation of cancer stem cells (CSCs) to relapse and therapy resistance. HA interacts with the CD44 receptor (a transmembrane glycoprotein involved in cell proliferation, migration, survival, and apoptosis) as well as intracellular signaling pathways, including that of tyrosine kinase. (**Figure 6**) These interactions promote the survival and proliferation of cancer cells (208). HA also influences mechanisms that regulate ATP binding cassette transporter expression, affects lipid metabolism in macrophages (209) and influences macrophage polarization (210), and HA regulates receptor tyrosine kinase pathways, with many important downstream affects. (**Figure 7**) Fascinatingly, HA can also defend against cancer development. Therefore, HA has a dual nature in tumorigenesis based on its molecular weight. Cancer resistance is seen with elevated high molecular mass (HMM-HA) production, in the absence of degradation (211) (reviewed in Schraverus et al. (212)). HMM-HA/HMW-HA (high molecular weight HA) has anti-inflammatory and immunosuppressive properties. It also regulates cell proliferation and migration, wound healing, and angiogenesis. Importantly, HMW-HA is an antibacterial, antiviral, and antifungal (83). Degrading HA into fragments (low molecular weight HA (LMW-HA)) induces the synthesis of inflammatory factors, including cytokines (77, 83, 213), modifies cell behavior and signaling (77, 83), and triggers angiogenesis, driving cancer progression (77). The equilibrium between the breakdown and synthesis of HA (the degradation balance) is controlled by hyaluronidases (HYALs) of which there are three HYAL classes in prokaryotes and five types in humans (214), CD44 (HA receptor), ROS, inflammatory factors, and HA synthase (HAS) (211). It is important to note that expression of CD44 is elevated in tumor-macrophages (204), which we theorize in cancer are highjacked by pathogens. The question arises of how does the degradation from high to lower molecular weight HA occur? Liu and colleagues (211) note that cells within tumors “hijack” HA production and fragmentation, and, thereby, promote cancer progression. Hyaluronan synthase 2 (HAS2) activity, which controls HA production, can be regulated by epigenetics, transcriptionally, or by post-translational modifications to control how much HA is produced (215) (**Figure 8**), which we theorize is important from the perspective of the pathogen and from the human immune system. We posit that not only is it the pathogens within the tumor that are hijacking the healthy cells and triggering the degradation of HA through genetic alterations but that some pathogens may be producing and using their own HA.

## 16 Pathogens produce hyaluronan

Bacteria and yeast both produce HA through fermentation (85). Varying the combinations of expressed genes and fermentation conditions control the yield and molecular weight of the HA that is produced by bacteria and fungi (84). Viruses are able to direct the host to synthesize HA by presenting the HAS gene. Pathogens that synthesize HA are highly virulent (83, 219). Bacteria and fungi that synthesize HA incorporate it into a mucoid capsule, which provides camouflage, protection against the immune system (220), and resistance to opsonization (83, 221), thereby avoiding being marked for elimination by phagocytes (221). Eliminating the mucoid capsule decreases virulence and increases the likelihood of phagocytosis. The concentration and molecular size of HA differs by human tissue type (83), and we theorize this may make certain organs more desirable for a pathogen than others in forming a tumor community.

Naked mole rats synthesize large amounts of extremely high molecular mass HA (EHMM-HA) of 6-12 MDa in brain and other tissues. The large amounts are due to the accumulation of EHMM-HA as a result of robust synthesis and slow degradation. The EHMM-HA that naked mole rats synthesize is substantially heavier (and more than five times larger) than the HMM-HA that humans synthesize (0.5-2 MDa) (82). Naked mole rats in the wild have been reported to not develop cancer (222). After removing EHMM-HA, naked mole-rats were able to develop malignant tumors (82). We postulate that EHMM-HA works as a strong antibiotic and that it is able to break up biofilms. Further, given the structural and mechanical abilities of EHMM-HA, we posit that it is also possible that the EHMM-HA produced by the naked mole rat and in such large amounts may provide physical barriers that keep out pathogens, hence, using our theory, protect against cancer.

## 17 CD44 expression and HA

CD44 is a cell adhesive molecule that interacts with the ECM component HA, which allows cells to adhere to their surroundings and facilitates cell migration. CD44 is also involved in angiogenesis by promoting migration of endothelial cells (216). Expression of CD44 is elevated in TAMs (204). LMW-HA attracts macrophages, which subsequently protect tumor cells from the immune system. This signaling for macrophages by LMW-HA is mediated by HA receptors, including CD44 (reviewed in Liu et al. (211)). Therefore, LMW-HA recruits macrophages that protect the cancer cells from the immune system (211) and promotes angiogenesis (216) and inflammation (77, 83, 213). We posit highjacked cells programmed by pathogens or the controlling pathogens form HMW-HA, which they can break down into LMW-HA to summon macrophages. Once macrophages are summoned, the pathogens use the HMW-HA to evade the immune system, and the pathogen (cancer) can spread and form distant tumors. CD44 is also overexpressed in cancer stem cells. CD44 facilitates cell-ECM and cell-cell interactions by connecting with HA. CD44+ colorectal cancer cells have both strong colony-forming and tumor-initiating capabilities (223). *Fusobacterium nucleatum* (*Fn*) has been associated with colorectal cancer, and high intratumoral loads are indicators of high risk of metastases, recurrence, and poorer patient outcomes (described in Zepeda-Rivera et al. (224)). When *Fn* infects cells, it leads to changes in CD44 expression and triggers cancer stem cell-like behavior, making these cells capable of forming tumors and migrating. *Fn* also may play a role in crosstalk between EMT and colorectal stem cells during the progression of colorectal cancer (223). EMT is a process whereby epithelial cells transform into mesenchymal-like cells through changes in cell adhesion, gene expression, and polarity. EMT can increase tumor invasiveness and metastasis (225). HA interactions with CD44 regulate cell survival and ERBB-family signaling, which are both important for tumorigenesis. The ERBB family of transmembrane proteins include ERBB1 (the epidermal growth factor receptor) and ERBB2 (HER2/NEU). Overexpression or mutation of ERBB1 and ERBB2 are often seen in breast, ovarian, and colorectal cancers (216). Various pathogens use ERBB1 for different roles, including entering into cells, suppressing host cell apoptosis, and inducing host cell proliferation (reviewed by Slanina et al. (226)). Some pathogens are able to bind to and activate ERBB2, which triggers certain signaling pathways that can contribute to the cellular entry of microbes and cancer (227). Entrance of pathogens into cells, we theorize, as well as increased cellular proliferation and lack of apoptosis, are fundamental aspects of tumors. Thus, we theorize upregulation of CD44 helps pathogens to infiltrate host cells and promote tumor formation by utilizing ERBB1 and ERBB2.

## 18 HA, the ECM, and tumor formation

The increase in production of HMW-HA, along with its fragmentation, supports our theory that pathogens are at the helm. Bacteria are able to fragment HMW-HA (228). Considering the capabilities of LMW-HA, the ability of bacteria (that can survive in phagocytes) to degrade HMW-HA would prove highly advantageous for survival and spread. Moreover, HA helps keep the tumor matrix from becoming rigid. When HA turnover is reduced below its norm, the matrix becomes rigid and the tissue becomes dysfunctional (229). That is from the human perspective. From the microbial perspective, the community becomes more protected. The ECM exerts regulatory control over signaling within the tumor, transport mechanisms, oxygenation, tumor metabolism, and immunogenicity. Hence, the ECM influences tumor growth and malignancy and its response to cancer treatments (reviewed in Henke et al. (76)).

## 19 Anti-cancer drugs are antimicrobials

**Figure 9:**
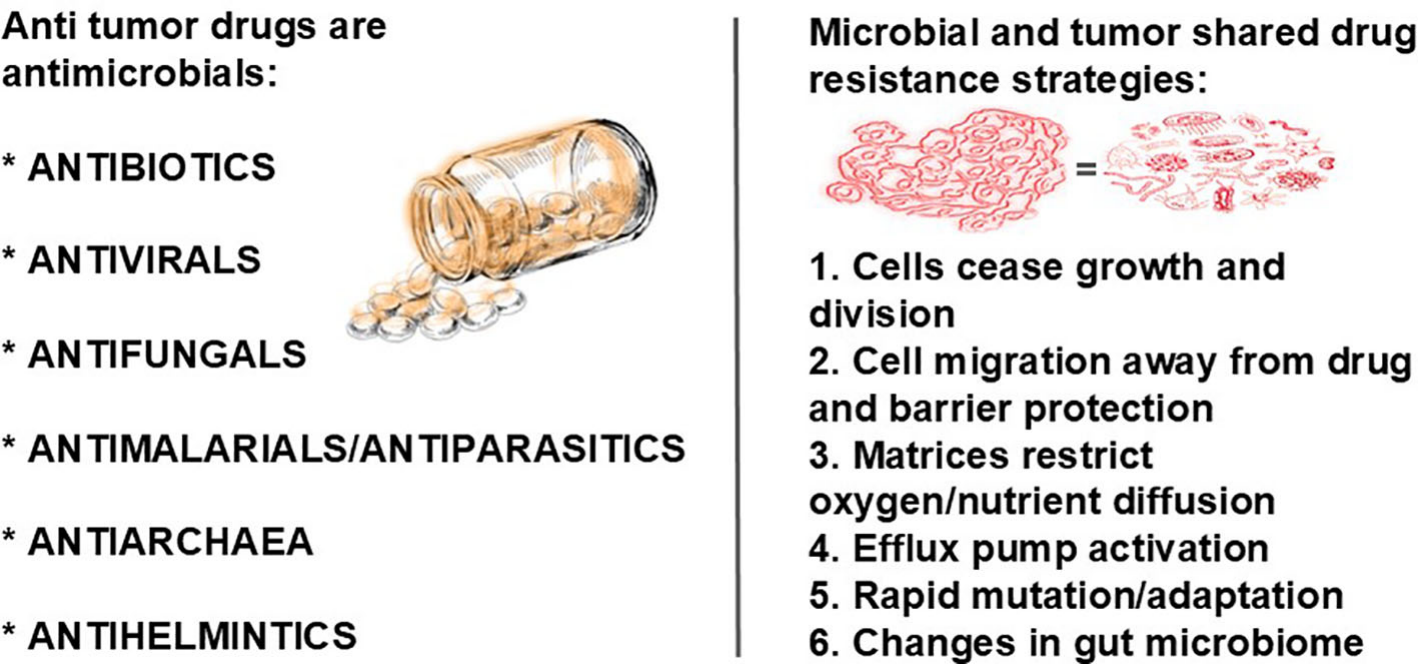
Anticancer drugs are antimicrobials, for example, Salinomycin, Mitomycin, Doxorubicin, Gemifloxacin, and Ciprofloxacin (230, 231), antivirals, antifungals, antihelminthics, and antimalarial/antiparasitic agents (230–232) and antimicrobials affecting archaea (233). A review by Pfab and colleagues (231) discusses anti-cancer activities exerted by antimicrobial agents, including antibiotics, antivirals, antifungals, antihelminthics, and anti-malarial/antiparasitic agents (230–232). Intratumoral microbial components within tumor tissues are closely correlated with therapy efficacy (234). Although not as widespread as antibacterial drugs, antifungal drugs are also used in cancer treatment (235, 236), as are antiviral drugs (232, 237, 238) and antiparasitic drugs (239, 240) as well as those effective against both cancer and archaea (233). Pathogens and eukaryotic tumor cells (which, according to our theory, are being controlled by pathogens) use similar drug-resistance strategies. Shared multi-drug resistance mechanisms by bacteria and cancer cells include efflux pump activation; adaptation (genetic mutations and horizontal gene transfer of genes showing resistance); and a collective stress response triggered by drug exposure intercellular communication of which there are at least six known strategies: (1) metabolic shifts in the absence of nutrients cause cells stop growing and dividing; (2) in response to drug administration, bacteria use their flagella to migrate to areas of low concentration and form biofilms to limit drug exposure, and tumor cells respond by metastasizing (which is also microbial movement, according to our theory) (reviewed in Chifiriuc et al. (241)) and developing a barrier vasculature (242); (3) the microbial biofilm exopolymeric matrix and the cancer cells’ stroma and ECM both restrict oxygen and nutrient diffusion and serve to protect both communities; (4) both communities use efflux pump activation in response to the presence of drugs; (5) both bacterial and cancer cells can increase mutation rates to accelerate evolution and, consequently, adaptation, making it possible for cancer cells and biofilms to quickly develop multi-drug resistance; and (6) both communities trigger changes in the gut microbiome that lead to drug resistance (reviewed in Chifiriuc et al. (241)).

Because many anti-cancer drugs are antimicrobials (**Figure 9**), as with increasing resistance to antibiotics, there is, increasingly, drug resistance in cancer (243). Cancer cells often become resistant to conventional anticancer antibiotics but highly sensitive to anticancer antibiotics in classes to which they have not been exposed (230). This describes the behavior of bacterial exposure to antibiotics (241). In addition to resistance, there are other reasons antibiotics are not always effective. Antibiotics inhibit or kill only multiplying bacteria but are not efficient in killing non-multiplying (metabolically inactive) bacteria. However, bacteria that are multiplying and those that are not multiplying co-exist in infections. Hence, non-multiplying bacteria are able to survive high concentrations of antibiotics. When exposed to ineffective drug concentration levels, the dormant bacteria can begin multiplying, re-infecting the host (244). This, we believe, explains why cancer returns after chemotherapy. In further support of this, it is known that dormant tumors and dormant cancer cells can be difficult to treat (245). Further, antibiotics do not treat microorganisms, other than bacteria, and complex tumor communities are composed of other microorganisms, as well.

## 20 The role of melanins in cancer

In 1963, Wassermann (120) found melanin granules in various immune system phagocytic cells. In 1977, Azzopardi and Eusebi (246) found, in the vast majority of cases, melanocyte colonization of breast carcinoma, where the dermal-epidermal interface had been encroached by tumor cells. “[T]he melanocytes exhibit in most cases this remarkable phenomenon of ‘climbing down’ into the mammary carcinoma.” The migration away from the epidermis to colonize the tumors left white patches in the epidermis, indicating a loss of melanocytes. However, the research team did not observe depigmentation in all of the tissue examined, and they suggest it is possible that melanocytes may proliferate while colonizing the tumor. Melanocytes were also found in groups of tumor cells within lymphatics. The pigmentation of the tumor cells can be seen only with certain staining techniques in the vast majority of cases. The authors describe, “The melanophages present in the region of the colonized carcinoma are the consequence of the ingestion of pigment liberated from colonizing melanocytes and from cancer cells” and that the “melanin pigment is dispersed as fine granules in the cytoplasm of the malignant cell.” We theorized and provided evidence supporting an inverse relationship between melanogenesis and ATP production, with melanin being the primary supplier of cellular energy via its intake of energy from light waves (20). Important to our theory is that the “colonization and pigmentation of breast carcinoma is associated with the presence of melanophages [immune cells involved in phagocytosis of melanin] in the vicinity.” The researchers, however, believed their presence alone was not important and so recorded those cases as a negative result. Hence, their findings reach 100 percent of cases from our perspective.

Melanocytes produce melanin and transfer the pigment to neoplastic cells. Melanophages are thought to result from ingestion by circulating macrophages of the pigment released from both colonizing melanocytes and from cancer cells (247). Epidermal-dermal interface disruption is thought to be required for melanocyte migration into the superficial dermis and superficial lymphatics to allow melanocytes to passively travel to regional lymph nodes by metastasizing tumor cells (246, 247). In one of two cases described by Santoro and colleagues, “The neoplasm showed a nevoid appearance with abundant melanin surrounding neoplastic cells with epithelioid morphology” (247). In the other, there was an area with “abundant tumor necrosis and no pigmentation” (247). Based on our theory, we would predict that the presence of melanin promotes tumorigenesis via a high energy supply and the absence of melanin would cause necrosis in the absence of upregulation of ATP or other source of cellular energy. Another way to see melanin’s role in cancer is to consider, again, malignant melanomas. Whereas malignant melanomas have melanin, benign nevi also have melanin. If melanin serves to absorb ultraviolet (UV) radiation to protect against gene mutation, then it would appear to us counterintuitive that UV radiation causes malignant melanoma. Yet increased eumelanogenesis is causally associated with malignant melanoma (7). Further, if malignant melanoma had only to do with no protection from UV radiation of the skin, then vitiligo would have a very high risk of malignancy, because it has no protection from UV radiation. With no melanin, which we propose is the primary fuel for cells (20), there are metabolic abnormalities in glucose and lipid metabolism and mitochondrial dysfunction seen in these skin cells (248). We proposed various melanins fuel cells (20), and increases in this fuel are necessary for cancer growth. We focus not only on eumelanin’s role in cancer, but also pheomelanin, as it can provide much more energy to cells (20). Indeed, there is evidence suggesting an increase in cysteine levels (cysteine is a precursor to pheomelanin) and pheomelanogenesis in malignant melanomas, and this also has been attributed to promoting genetic mutations, rather than to eumelanogenesis (7). We conclude that pathogens, energized by melanin, especially pheomelanin, the production of which is increased with UV radiation, cause the genetic mutations that lead to cancer and that melanin fuels other oncogenic processes.

It does not appear through literature searches that much consideration has been given to melanin and cancer outside of malignant melanoma. As noted above, there has been some research on breast cancer cases, which are the next most frequently described cancer where melanocyte colonization has been observed, and there are several other tumor types also seen (for example, Nestor et al. (249), Modica et al. (250), Gough and Benediktsson (251), Waxman et al. (252)) when using various staining techniques (251). However, there has been interesting research in cysteine and the immune system as it pertains to cancer. Myeloid-derived suppressor cells (MDSCs) hinder the immune system’s ability to fight cancer by hoarding cystine and preventing T cells from getting the cysteine they need to become active to mount an effective immune response against tumors. It is also interesting to note that MDSCs compete with macrophages and dendritic cells, both antigen-presenting cells, for cystine. Another study showed that tumor mesenchymal stem cells can sequester cysteine away from dendritic cells. Dendritic cells cannot synthesize cysteine effectively. As a result, dendritic cells are unable to provide cysteine to naïve T cells (reviewed in Nin et al. (175)). We understand this as suggesting that phagocytes, which we theorize house pathogens, use cysteine, hence, pheomelanin, as fuel, and that pathogens block cysteine, hence, pheomelanin, the energy source, from these tumor suppressor T cells. In yet another study, cysteine deprivation stopped ovarian clear cell carcinoma growth (253).

## 21 Pheomelanin fuels tumor growth and metastasis

**Figure 10:**
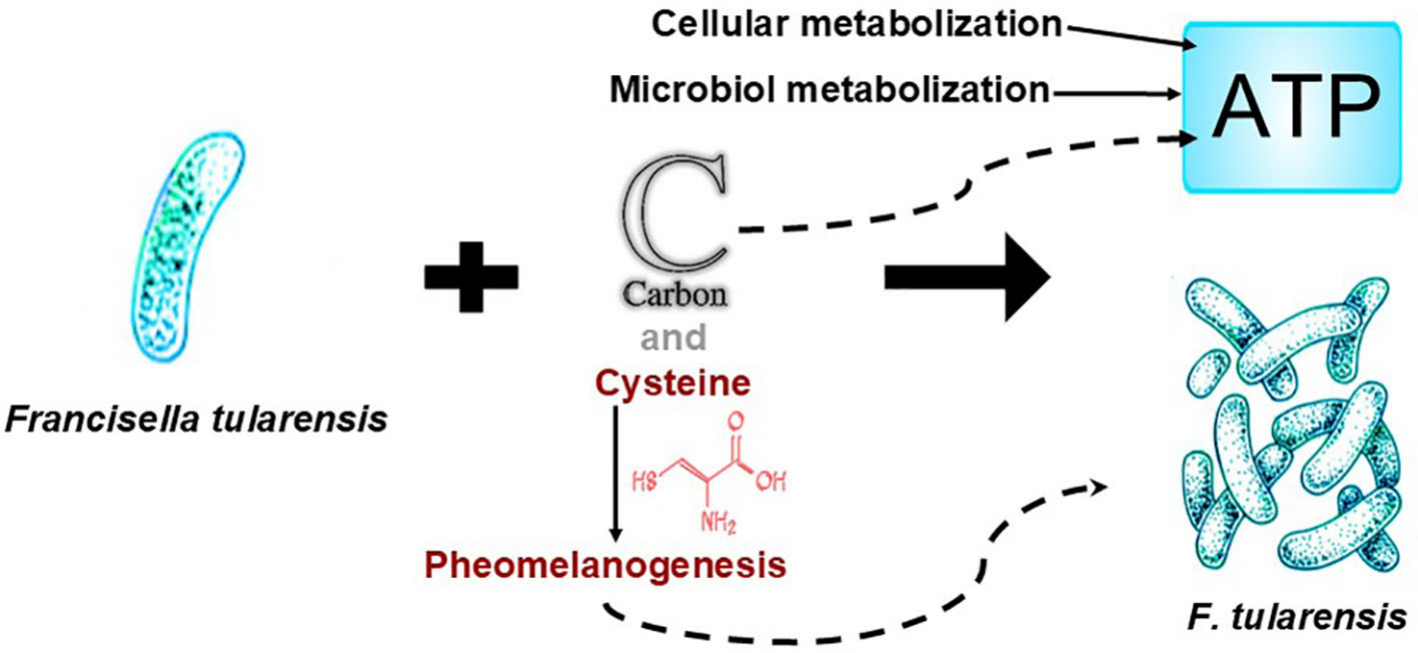
Cysteine is required for intracellular replication of *Francisella tularensis*, a cytosolic IBP, along with host-derived carbon sources (257). This suggests to us that other carbon sources, which include almost all known cancer risk factors, can be used by pathogens via cellular metabolism and/or microbial metabolism for conversion into ATP or melanin and that cysteine may be being used as a precursor to pheomelanin, which we theorize is an important underlying energy source for pathogen replication, and, hence, tumors.

Melanogenesis results in both eumelanin and pheomelanin in various ratios. The ratio (and, hence, color) is determined by the level of tyrosinase activity and tyrosine and cysteine concentrations (254). Cysteine levels determine if dopaquinone enters the pheomelananogenesis pathway or eumelaninogenesis pathway. High cysteine levels produce more pheomelanin, and low cysteine levels produce more eumelanin. Interestingly, there is a competitive behavior between the two reactions (255), which suggests to us a switch in various energy level needs, and is of great importance to our unifying theory of disease in which we show the inverse behavior between melanogenesis and ATP production (20). Mitra and colleagues (256) investigated the eumelanin/pheomelanin ratio impact on mouse models used to mimic human phenotypes and albinism (no melanin) and found without additional gene aberrations or UV radiation exposure, red fur mice (pheomelanin being predominant in red fur/hair) exhibited a high incidence of invasive melanomas, and the absence of pheomelanin synthesis in the genetically modified albino mice was found to be protective against melanoma development. They concluded that UV radiation is not needed for pheomelanin to be carcinogenic and that pheomelanin, itself, may be carcinogenic. We do not believe that pheomelanin is carcinogenic. Rather, as noted previously, we theorize that the energy it contains is being used by the pathogens in the tumor and to fuel the tumor cells, so we would predict that pheomelanin introduced into a mutated cell would fuel its replication. (**Figure 10**) Further, Mitra et al. note that some melanomas develop in areas that are not exposed to sunlight. However, photons travel through matter. We also theorize that pheomelanin is being used by microbes to fuel angiogenesis. One indication is that individuals with lighter skin have more diseases related to angiogenesis compared to darker skinned individuals (258), and individuals with lighter skin have more pheomelanin than individuals with darker skin (259). In order for either of these theories to be possible, there must be evidence of its precursors at increased levels within tumors, and, indeed, it appears that both amino acids cysteine and cystine, the latter of which is formed when two cysteine molecules combine, are found in tumors and are associated with tumor formation, propagation, and treatment resistance. In fact, there is increased cysteine and cystine uptake in tumors (reviewed in Nin et al. (175)). Pathogens are known to produce cysteine (260), alter cysteine availability in their human hosts, and utilize melanin produced by their host, which we detailed previously (20). Therefore, we theorize that pheomelanin production is controlled by pathogens and used as their primary source of high energy. We theorize that it may be that tumor communities use eumelanin in higher ratios to pheomelanin for homeostasis and slow growth and pheomelanin for more rapid growth and aggressive metastasis, as evidenced by dark melanotic melanomas, which are brown/black (eumelanin) versus amelanotic melanomas, which are pink (and we postulate contain pheomelanin) and much more aggressive (261). Indeed, Sarna and colleagues (262) found melanin pigmentation to be highly deregulated in melanoma cells, which can switch between pigmented and non-pigmented states. They also found that livers from mice that were inoculated with non-pigmented melanoma cells formed more metastatic tumors compared with mice inoculated with pigmented melanoma cells, and the non-pigmented tumors were heavier in comparison.

## 22 Tumor cells and TAMs scavenge cysteine from the ECM for pheomelanogenesis

Both eumelanin and pheomelanin synthesis begin with phenylalanine and both move through the same pathway until they split at dopaquinone. It is at that point that the pathway either moves into L-DOPA to eventually form eumelanin or it moves to cysteinyldopa, requiring cysteine, for production of pheomelanin, which we previously discussed (20). Cysteine availability above 0.13 μM causes the shift from eumelanin production to pheomelanin production (263). Therefore, it is of great interest that cathepsin B, a lysosomal cysteine protease that is found in most cell types but is most abundant in macrophages (264) and upregulated in tumor cells and TAMs (265, 266), is able to remove the amino acid cysteine from proteins (264, 267) and that bacteria that infect macrophages intracellularly are able to regulate the amount of cathepsin B produced by the infected macrophages and are able to affect the trafficking of cathepsin B (264). Therefore, we theorize that intracellular pathogens regulate the amount and trafficking of cathepsin B in tumor cells. In fact, tumor cells and TAMs release cathepsin B into the extracellular matrix, where it breaks down the components of the extracellular matrix by removing cysteines from these components (summarized by Larionova et al. (268)). We theorize that tumor cells are controlled by microbes and scavenge the cysteines that the released cathepsin B proteins break off from the extracellular matrix. Indeed, system x_c_^−^, a transporter that uptakes extracellular cystine into the cell, has been found to be upregulated in cancer (269). In the oxidizing extracellular environment, free cysteines combine to form cystine (270), and, thus, releasing cathepsin B into the extracellular matrix would create an abundance of extracellular cystine, which we theorize the tumor cells then uptake and reduce back to cysteine to produce pheomelanin to provide increased energy to the tumor cells, which the tumor cells then use to survive, proliferate, and metastasize. In support of our theory, it is known that the release of cathepsin B into the extracellular matrix leads to tumor cell proliferation and invasion (summarized by Larionova et al. (268)). We further theorize that this process provides additional protection for the tumor, as an increase in uptake of extracellular cystine by tumor cells and TAMs for pheomelanin production deprives T cells and NK cells of cysteine, which is necessary to produce glutathione, impairing NK cell and T cell function (271). As noted in **Table 1**, NK cells are antitumor and T cells are generally antitumor.

Cathepsin B, implicated in both tumor invasion and metastasis across various cancers (265), is notably overexpressed in cancers with MYCN gene amplification (272). The protein N-Myc, encoded by the MYCN gene, is associated with an elevated release of proteins, including cathepsin B, which in turn appears to enhance the invasiveness of cancer cells and their resistance to therapies. Inhibition of cathepsin B has been shown to curb the migratory behavior of these cells and increase their susceptibility to the chemotherapeutic agent doxorubicin (272). Additionally, the concept of “cysteine addiction” in cancer is linked to the MYCN gene; cysteine depletion triggers extensive lipid peroxidation (273), suggesting its pivotal role in MYC-driven cancer processes. We theorize it is fueling the cancer microbial and fungal communities. Without it, there is cellular damage and destruction (273).

## 23 Mitochondria in tumors

**Figure 11:**
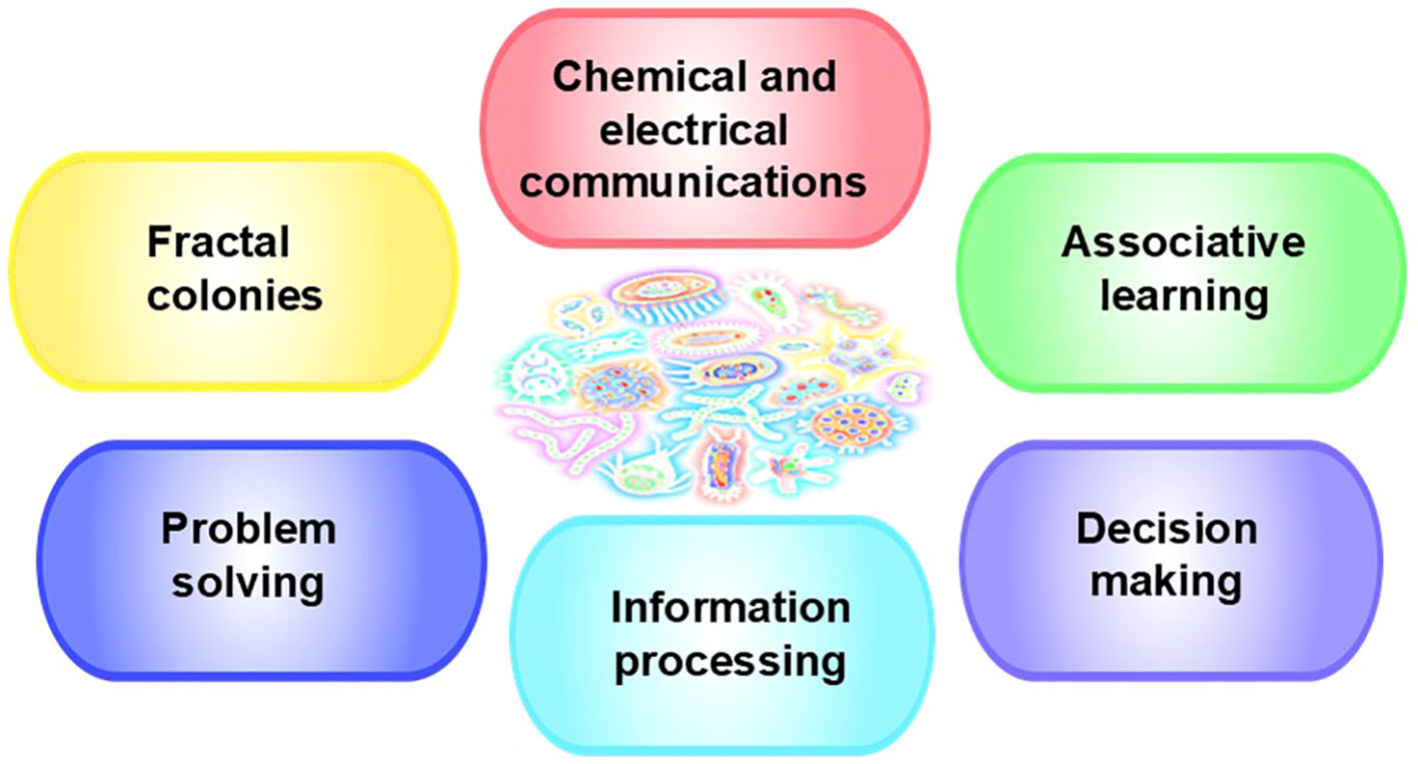
Indicators of microbial intelligence: Fractal colonies: Bacteria form patterned colonies, most notably, fractals. Bacteria utilize sophisticated cooperative behavior and intricate communication to self-organize, the latter both at the singular bacterial cell and colony levels, in determining and in building out the patterns (277). Fractal patterns, made of bacteria biofilms and pellicles (soft biofilms formed at the air-liquid interface) from heterogeneous micro colonies, increases surface area, enhances protection from drugs and the immune system, enhances signal transduction, promoting collective behaviors, promotes access to nutrients (278), and promotes faster biofilm growth to quickly obtain nutrients in an environment lacking nutrients (279). Presumably, the increased surface area of fractals allows an increased number of bacteria to have access, enhancing the absorption of nutrients and the expulsion of waste products and increasing the local proximity of motile to matrix cells, which may facilitate the exchange of signaling molecules, nutrients, and genetic material, promoting cooperative behavior and resource sharing among cells. Bacteria must navigate complex topologies, including mazes or fractals. Phan and colleagues (280) showed how *E. coli* moved through nontrivial mazes in significantly shorter times than predicted by a no-memory walk and demonstrated how they can collectively leave a fractal. Strategies observed include bet hedging, used to avoid nutrient deprivation, if the riskier attempts at finding food failed. Mycelium in soil also grow in fractals (281). Therefore, it is of importance to note that cancer cells, too, display fractals and researchers have used quantifying “fractalness” along individual cell borders to distinguish between types of cancer with 97 percent accuracy (282). We theorize that within each cancer cell there exists microbial communities, also fractals, hijacking those cells. Chemical and electrical communications: Bacterial communications take the form of quorum sensing and electrical signaling, among other forms of communication. Fungi also use chemical signaling in quorum sensing (283), intraspecies and interkingdom communications (284), and network electrical signaling (285). Problem solving: Upon cues indicating phosphate depletion in the intestinal tracts of patients following surgery, *Pseudomonas aeruginosa* can shift from a benign colonizer to lethal pathogen (286), presumably to escape to an environment with a food supply. Phosphate is an essential nutrient for *P. aeruginosa* (287) and is abundant in soil (288). *Physarum polycephalum* can determine the shortest path between two food sources in a labyrinth to maximize its foraging efficiency (289). Fossil evidence shows for the last 48 million years the fungus *Ophiocordyceps unilaterali*s has been infecting foraging ants with its spores, which, once colonized internally, hijack the central nervous system of the ant, forcing it to transport the spores to higher ground and latch onto vegetation, where the fungus kills the ant, grows, and releases spores. To accomplish this, the fungus uses various metabolites, depending on ant species, to mediate ant interactions with ant tissue, depending on the specific ant species’ brain. The fungus grows nearby the brain and manipulates ant behavior by secreting a sphingosine, a metabolite that is part of sphingolipid metabolism affecting cell regulation (290). Sphingosines are involved in cancer, including breast cancer, colon cancer, gastric cancer, prostate cancer, pancreatic cancer, and other cancers (291). Importantly, most bacteria and viruses that are not able to produce sphingolipids are able to use that of their host to promote virulence, and in the cases of protozoa and fungi, both the pathogen and host produce and use sphingolipids. Sphingosine-1-phosphate has been shown to polarize macrophages toward an M2-like phenotype (292, 293). We believe *O. unilateralis* controlling the brains of foraging ants using a metabolite serves as a useful model of how pathogens are able to take over the control of cells in cancer, neurogenerative diseases, cardiovascular diseases, and other diseases. Decision making: Decision making in microorganisms is commonplace, as is seen in communication (signaling pathways), changes in gene-expression, purposeful movement, and other behaviors. Similar to microorganisms, fungi also use communications, changes in gene expression, purposeful movement, and other behaviors in response to stimuli. They also have memory (294). Fungal mycelia demonstrate decision making and consequential behavior by changing their developmental patterns in response to other organisms and may have spatial recognition (summarized in Money (295)). Fungi decision-making abilities are made clear in their negotiation skills: Mycorrhizal fungi act as shrewd negotiators in resource exchange. These fungi trade phosphorus with tree roots in exchange for carbon. Specifically, fungi growing in resource-poor patches showed higher trade gains, with increased fungal biomass per unit of phosphorus transferred compared to those in resource-rich patches. This suggests that as phosphorus availability decreases, its net value increases. Consequently, the fungi move phosphorus from rich patches, where it has a lower value, to poor patches, where it has a higher value. In the absence of resource inequality, there was no difference in the exchange rate (296). Associative learning: Linking two events together and acting upon the outcome is present in microorganisms and fungi, as seen in the above problem-solving examples. Information processing: Building and navigating fractals, communication, associative learning, problem solving, and decision making all require information processing, which is present in both bacteria and fungi.

Researchers have recently found that the Warburg effect (274) is not fully accurate. The mitochondria in tumors generally remain healthy and in a working state (275). In fact, researchers have found that cancer cells build an arsenal of mitochondria by stealing them from T cells, weakening the host’s immune system while building the tumor’s energy supply. Saha and colleagues (276) observed breast cancer cells sending out nanotubes, tube-like filaments, to T cells. These “tentacles” pulled the mitochondria out of the T cells, which travelled down the nanotubes and were incorporated into the cancer cells. Analysis of the metabolic functions of both cells showed the taking of mitochondria affected cell function (276). This is also another example of what appears to be “intelligent” behavior (**Figure 11**).

## 24 Drugs affect melanogenesis

The current understanding of cancer is that inflammation is carcinogenic (297). We argue here that inflammation is a consequence of pathogenic invasion and that the reason some NSAIDs are anticarcinogenic is that they reduce melanogenesis, which we theorize is fueling tumorigenesis. (Some NSAIDs increase melanogenesis, as can be seen in hyperpigmentation side effects, and we postulate that they may fuel cancer, if it is present). For example, aspirin is an anti-inflammatory, has anti-tumorigenesis properties, and inhibits melanogenesis (297). Indeed, in addition to aspirin, certain other NSAIDs have been associated with a reduction in cancer risk (297), for example, breast cancer (298, 299), prostate cancer (300, 301), ovarian cancer (302), colorectal cancer (303, 304), and head and neck cancers (305), and Celebrex (celecoxib), an NSAID, enhanced the effect of trametinib, for example; both inhibit tumor-associated melanogenesis (306). There is some research that *appears* to contradict these findings. However, those drugs are not anti-melanogenic. Some drugs cause hyperpigmentation, and we would predict that these drugs would, in fact, promote cancer. Interestingly, aspirin has an inhibitory effect on MITF via two pathways, which, as a consequence, inhibits tyrosinase, which is responsible for catalyzing the reaction critical to the formation of melanin (307), as our theory that melanin is fueling cancer would predict. Aspirin and celecoxib were found to reduce (tumor) colony formation and cell motility (as well as pigmentation) via a different pathway (via suppressing PGE2 and activating AMPK) in another study (308), further supporting our theory on melanin and cellular energy. Aspirin downregulates homocysteine production (309). Homocysteine can be converted into cysteine (310). Cysteine is a precursor to pheomelanogenesis. Pigmentary changes have been reported in as high as 75 percent of individuals treated with targeted anticancer agents (311). Nicotine also affects melanin levels (20). Tamoxifen, and presumably other chemotherapies, work differently but also inhibit melanin production. In ER+ breast cancer, the cancer cells have high levels of estrogen receptors and are particularly sensitive to estrogen. These receptors are proteins that bind to estrogen, which circulates in the body at normal levels. When estrogen binds to these receptors, it can stimulate the cancer cells to proliferate. Estrogen has been found to play a significant role in the regulation of melanin synthesis. This effect is mediated through nonclassical membrane-bound receptors known as G protein-coupled estrogen receptors (312). Tamoxifen is an ER blocker (313). In blocking estrogen from breast tissue, we theorize it is also inhibiting melanogenesis locally.

## 25 NSAIDs and other anti-cancer drugs target macrophages and other phagocytes

An important part of our theory is the role that phagocytes play in tumorigenesis and metastasis. We have previously described in this paper that anti-cancer drugs are antimicrobials. We have also discussed that it is likely that bacteria control not just their own synthesis of melanin but also that of their host, or at least use the melanin that their host produces, and that aspirin and certain NSAIDs affect melanogenesis. It is also important to note that aspirin and other NSAIDs also affect macrophages and other phagocytes. Their effects are dose dependent and also dependent on the specific NSAID (314). In addition to reducing melanin, aspirin increases phagocytic uptake by macrophages (315); however, aspirin causes macrophages to shift polarization from M2 to M1 (316). This is evidence that aspirin frees the macrophages from being hijacked by pathogens. Thus, we would theorize that aspirin would have anticancer effects, and, indeed, it does (317). It also has been shown to prevent metastases (318). However, not all NSAIDs have the same targets. Phenylbutazone was found to increase macrophage phagocytic uptake up to two-fold (315), and, not surprisingly according to our theory, phenylbutazone has been found to cause cancer in mice and rats (319). Further, some NSAIDs do not affect phagocytes (315). We believe all these variables – whether or not an NSAID affects phagocytes and in what ways and how and whether an NSAID affects melanogenesis – account for the variability in outcomes seen in research on NSAID use and their effects on cancer. Finally, traditional anti-cancer drugs also affect phagocytes (320–322). We believe the significance of our discovery of these linkages in the context of our theories cannot be overstated.

## 26 Common risk factors are really forms of energy

Many risk factors have been associated with cancer: tobacco use, diet (fried foods, red meat), being overweight/obesity, physical inactivity (which can be seen as a buildup of various energy stores), drinking alcohol, indoor and outdoor pollution, UV radiation, and carcinogens in the workplace (323). We theorize these risk factors are used as energy sources by certain microorganisms to carry out their tumor-building functions, and, therefore, can trigger cancer when there is enough energy and the presence of microbes that can utilize those molecules (which can explain why people exposed to the same risk factors do not all develop cancer). In fact, Nejman and colleagues (46) investigated the functional activities of intratumor bacteria and found preferred niches by tumor type. For example, in non-small-cell lung cancer, there was a high prevalence of heterogeneous bacteria that are able to utilize the chemicals from cigarette smoke metabolites and biosynthesize metabolites used by plants which, the authors speculate, may have come from the tobacco plants. They had similar findings in breast cancer subtypes. In ER+ breast tumors, which have increased oxidative stress compared with ER- breast tumors, they found enriched pathways in bacteria for arsenate detoxification and mycothiol biosynthesis. Arsenic exposure is a risk factor for this subtype of breast cancer, and bacteria have been shown to use mycothiol to detoxify ROS. The team hypothesized that bacteria that could synthesize mycothiol had better survival rates in the ER+ tumor microenvironments. As they note, their analysis of pathways used by bacteria suggests that the tumor environment is associated with bacteria that have functions that survive well in the tumor microenvironment (summarized in Nejman et al. (46)).

The connection of cancer risk factors to microbial energy and cancer development is not always easy to make. Asbestos, a known carcinogen (324), is an intriguing example. While it is commonly thought that asbestos causes lung cancer due to its fibers causing inflammation (324), we theorize it is due to bacteria feeding off of iron contained in asbestos fibers. Indeed, most microorganisms that live in soil rely on iron to generate energy. Certain fungi use the iron found in crocidolite, a highly carcinogenic form of asbestos. Without this iron, researchers demonstrated that the fibers were unable to generate the free radicals associated with causing cancer (325, 326). Bacteria also have been shown to act similarly to fungi in using the iron in asbestos (summarized in Choi et al., (327)). We postulate that not only can asbestos feed existing pathogens, but when individuals inhale asbestos, the fibers are already contaminated with microorganisms feeding off the iron.

We found no evidence in a literature search that methods for *in vitro* experiments designed to determine the effects of radiation on animal or human cells in terms of mutation outcomes include sterilization of cells prior to radiation. Therefore, it remains unknown if radiation is energizing pathogens, and the pathogens are causing the mutations, or if the radiation is directly causing mutations. This also brings up the question of if radiation therapy in cancer works by killing pathogens. Further, in a literature search, we saw no research to determine if in cases where radiation therapy is not effective, if pathogens are present in the surviving cancer cells. We strongly suggest research in these areas.

## 27 Testing the theories

We provide strong evidence that tumors are complex communities composed of various microorganisms. Therefore, using only an antibacterial or only an antiviral or only another species-specific drug may not fully treat cancer. Effective treatments may require examination of biopsies and areas surrounding the tumor, blood and lymphatic systems, and certain immune cells for pathogens, including fungi, and their markers, and a combination of targeted antimicrobial drugs. We suggest investigating intratumoral administration of antimicrobial drugs for localized, early-stage solid tumors and combination therapies of IV antimicrobials combined with drugs effective in penetrating biofilms for more advanced tumors. Pathology of any excised tumor tissues should include multiple staining types, especially for fungi, and samples should include multiple locations of each mass due to variation in microbial communities throughout the tumor. In order to identify these complex biomes, DNA sequencing should be performed. Therefore, we suggest routinely thoroughly examining tumor specimens and surrounding areas for microbes. Because we theorize that benign tumors are relics of a war between commensal and pathogenic microbes, we suggest further research on the use of commensal microbes, especially commensal bacteria, in the treatment of tumors, particularly early-stage tumors and any other “enemy” microbes. Further, probiotics, prebiotics, microbial toxins, metabolites, small molecules, and certain immune cells as well as anti-platelet drugs should be investigated as adjuvants.

We suggest testing for and eliminating any phagocytes in stem cell and bone marrow transplantation to prevent cancer recurrence.

One remarkable antimicrobial that we suggest is worthy of investigation is the EHMM-HA derived from naked mole-rats (*Heterocephalus glaber*) due to its antimicrobial potency and its ability to break up biofilms, which may have the added benefit of preventing, and possibly, removing plaque, which we postulate is part of the tumor.

Fungi are present in tumor microbiomes. However, not all fungi are tumor promoters and some are anti-tumoral, for example, *Trametes versicolor*, also known as turkey tail mushroom. It has antimicrobial properties. We suggest further investigations into this and other fungi as well as microbes that would offset growth of various tumor microbial and fungal communities.

We also suggest investigating the use of drugs, natural chemicals, for example, phytoncides, and technologies that would trigger immune responses by non-phagocytizing immune cells, such as natural killer cells, as is the case for phytoncides. For certain cases, the possibility of phage therapy and/or plasmid therapy should be investigated.

Finally, we are most intrigued by the potential of photobiomodulation. It is possible that light at wavelengths and intensities tailored to target a specific tumor pathogen community could penetrate into the tumor, kill the pathogens, and reduce inflammation. Of course, it is important to avoid exposure to wavelengths of light that would trigger melanogenesis, especially pheomelanogenesis.

In the future, after research determines what the microbiome makeup is of specific tumors, we suggest consideration of ordering antibody titers to common tumor pathogens during annual checkups and administering antimicrobials, if indicated, to prevent the development of cancer.

### 27.1 A cautionary statement

While we theorize that melanin is an important part of the immune system and vital for cellular energy, we caution against using any treatment that increases melanin prior to eradicating pathogens, because an increase in melanin might fuel the pathogenic process.

## 28 Conclusion

We theorize that cancerous tumors are complex microbial communities composed of various microorganisms living within biofilms encapsulated by a hard matrix. We found intriguing evidence that pathogens evade the immune system and spread by hiding within immune system cells and traveling inside them to distant sites where they form more tumors (metastasize). Because we realized that pathogens could hide and survive within immune cells that phagocytize, we further investigated this observation and concluded that immune cells that phagocytize are protumor and, as evidence, when there are large numbers of them in the tumor environment, the prognosis is poor. In contrast, immune cells that do not phagocytize are anti-tumor, and when there are large numbers of them in the tumor, the prognosis is improved. Genetic changes that trigger the activation of oncogenes or the inactivation of tumor suppressor genes is the most common cause of tumorigenesis (198). We theorize that it is pathogens inactivating or activating genes, that pathogens utilize melanin for energy in building and sustaining tumors and in metastasis, and that pathogen-hijacked cancer cells/tumors evade the immune system and travel to distant sites using phagocytes and platelets.

Bacteria and other microorganisms, which have been around for billions of years, have evolved mechanisms to manipulate host cell processes to create more favorable environments for their survival and proliferation. Understanding how microorganisms work, alone and in synchrony, pathogenic and commensal, will help us better understand cancer. Changing perspective from understanding cancer as a host self-cell aberration disease to viewing the host as an environment where microorganisms manipulate cell genetics and functions for their own advantage is of paramount importance. The significance of our discoveries in the framework of our theories cannot be overstated, if we are to effectively treat, and one day prevent, cancer.

## Data Availability

The original contributions presented in the study are included in the article/supplementary material. Further inquiries can be directed to the corresponding authors.
